# Versatile Nanoparticle Capsule Formation With Enhanced Encapsulation Efficiency via Solute‐Induced Liquid–Liquid Phase Separation

**DOI:** 10.1002/smll.202502573

**Published:** 2025-05-07

**Authors:** Takehiro Yachi, Honoka Watanabe, Rumi Niwa, Daisuke Unabara, Tasuku Hamaguchi, Yusuke Yonamine, Koji Yonekura, Kuniharu Ijiro, Hideyuki Mitomo

**Affiliations:** ^1^ Research Institute for Electronic Science Hokkaido University Sapporo 001‐0021 Japan; ^2^ Graduate School of Life Science Hokkaido University Sapporo 060‐0810 Japan; ^3^ Graduate School of Life Sciences Tohoku University Sendai 980‐8577 Japan; ^4^ Institute of Multidisciplinary Research for Advanced Materials Tohoku University Sendai 980‐8577 Japan; ^5^ Biostructural Mechanism Laboratory RIKEN SPring‐8 Center Sayo Hyogo 679‐5148 Japan

**Keywords:** enhanced encapsulation, inorganic nanoparticles, nanoparticle capsules, pickering emulsion, self‐assembly

## Abstract

Nanoparticle capsules (NCs), capsule‐like structures composed of inorganic nanoparticles (NPs), hold great potential for diverse applications due to their structure‐driven functionality and the unique properties of the NPs. Despite advancements in NC formation methods, achieving both stable formation and efficient material encapsulation remains challenging. In this study, a simple and versatile method is developed for the formation of inorganic NCs. Inorganic NCs are formed from oligo(ethylene glycol) (OEG)‐modified inorganic NPs via self‐assembly at solute‐induced liquid–liquid interfaces. First, Au NCs are formed from OEG‐Au NPs. Their formation is confirmed by scanning transmission electron microscopic (STEM) and cryo‐electron tomographic (ET) observations. The resulting Au NCs exhibited high stability, uniform size, and precise tunability under various conditions. The method is further extended to magnetic Fe_3_O_4_ NPs, affording size‐controlled magnetic NCs, demonstrating broad applicability. Moreover, this approach enables the efficient encapsulation of various materials, including citrate‐capped Au NPs and DNA molecules, into inorganic NCs, affording >2000‐fold enrichment. This method paves the way for innovative application of inorganic NCs in drug delivery, nanoreactors, and beyond.

## Graphical abstract

A simple and versatile method is developed to form inorganic nanoparticle capsules (NCs) via self‐assembly at solute‐induced liquid–liquid interfaces. This approach enables the stable formation of size‐tunable NCs from oligo(ethylene glycol)‐modified inorganic nanoparticles. Additionally, efficient material encapsulation is achieved with a 2000‐fold enrichment. This method opens new possibilities for diverse applications of inorganic NCs.

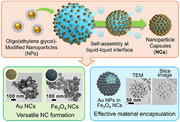

## 1 Introduction

Nanocapsule structures are attracting attention due to their potential applications in various fields, such as drug delivery systems (DDSs) and nanoreactors that utilize internal spaces. Typically, organic capsules such as liposomes have been extensively studied as DDS carriers,^[^ ^1^, ^2^, ^3^, ^4^, ^5^, ^6^ ^]^ as well as in relation to cosmetics^[^ ^7^, ^8^, ^9^ ^]^ and food applications,^[^ ^10^, ^11^ ^]^ owing to their biocompatibility, high encapsulation efficiency, and low toxicity. In DDSs, drug release can be controlled by designing liposomes to respond to environmental changes such as pH^[^ ^12^, ^13^ ^]^ and temperature,^[^ ^14^, ^15^ ^]^ generating considerable interest in their potential applications. However, organic molecules have relatively low responsiveness to external stimuli such as near‐infrared light and magnetic fields, which are permeable to living organisms. This limitation restricts their use in stimulus‐triggered drug release. In addition, the physicochemical fragility of organic nanocapsules further constrains their practical applications.

In recent years, nanoparticle capsules (NCs) composed of inorganic nanoparticles (NPs) have attracted significant attention. Inorganic NPs are known to exhibit unique size‐ and shape‐dependent properties that differ from bulk materials, including plasmonic NPs, quantum dots, and superparamagnetic NPs. These distinctive properties can be leveraged in the design of inorganic NCs. In addition, as inorganic NCs possess a relatively flexible structure that can be controlled by assembly‐disassembly, the encapsulation and release of substances are expected to be controlled, similar to organic nanocapsules. Therefore, inorganic NCs hold promise for a wide range of applications, including DDSs,^[^ ^16^, ^17^, ^18^ ^]^ bioimaging,^[^ ^16^, ^17^ ^]^ surface‐enhanced Raman scattering (SERS),^[^ ^18^, ^19^, ^20^ ^]^ and nanoreactors,^[^ ^21^ ^]^ through the utilization of both the material properties and capsule structures.

To date, several methods have been reported for inorganic NC formation. As a representative method, NC formation by self‐assembly of polymer‐modified NPs has been reported.^[^ ^16^, ^18^, ^22^, ^23^, ^24^, ^25^ ^]^ Inorganic NPs modified with either block copolymers containing hydrophobic and hydrophilic domains or a combination of hydrophobic and hydrophilic polymers undergo self‐assembly to form vesicle structures. This method offers advantages in terms of stable vesicle formation and enhanced dispersion stability of NCs. Indeed, the formation of NCs from various inorganic NPs, including anisotropic nanoparticles, has been reported,^[^ ^26^, ^27^, ^28^ ^]^ and their applications as DDS carriers have also been investigated.^[^ ^16^, ^17^, ^22^, ^23^ ^]^ However, the complexity of polymer design and challenges associated with efficient substance encapsulation remain significant obstacles. In material encapsulation, in particular, the concentration of the target material inside the NCs is determined by its concentration in the solvent used for NC formation, and a significant portion remains outside the NCs, resulting in considerable losses. Pickering emulsions, which are stabilized with fine particles, are another promising strategy for forming capsule structures from particles. They can be easily prepared by sonicating an oil/water mixture with fine particles.^[^ ^20^, ^29^, ^30^, ^31^ ^]^ In addition, differences in solubility between the inner droplet and the outer solvent enable the efficient encapsulation of molecules into NCs. Most Pickering emulsions are of micron order,^[^ ^31^, ^32^, ^33^, ^34^ ^]^ whereas nanoscale capsule structures are required for efficient cellular uptake in DDS carriers^[^ ^35^, ^36^, ^37^ ^]^ and the void‐confinement effect in nanoreactors.^[^ ^38^, ^39^ ^]^ On the other hand, nanoscale capsule structures are unstable and prone to collapse due to coalescence. Several studies have demonstrated that fixing the structure improves the stability of even nanoscale NCs;^[^ ^30^, ^40^ ^]^ However, these approaches complicate the NC formation process, and rigid, inflexible NC structures limit their potential applications, such as drug release. Therefore, achieving stable and nano‐sized inorganic NC formation through Pickering emulsions remains a significant challenge.

**Scheme 1:**
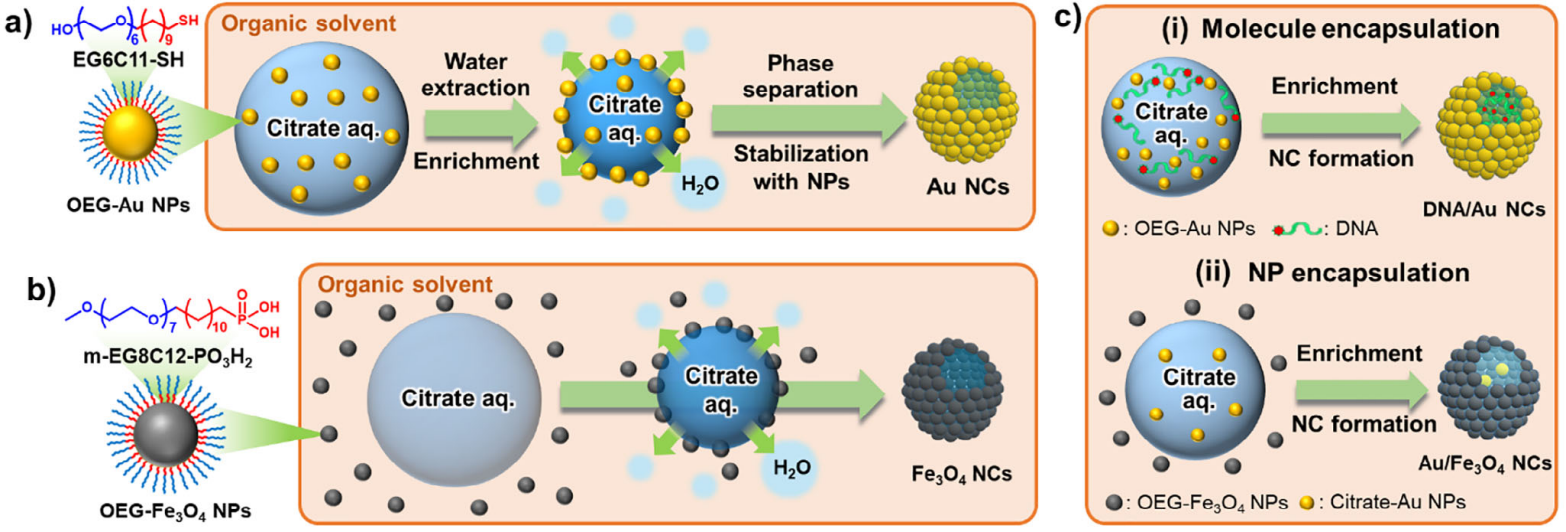
a) Formation mechanism of Au NCs via self‐assembly of OEG‐Au NPs at the solute‐induced liquid–liquid interface. b) Formation mechanism of NCs from OEG‐Fe_3_O_4_ NPs dispersed in organic solvent. c) Schematic illustration of the effective encapsulation of (i) molecules and (ii) NPs into NCs.

Our previous study also found that Au NCs can be formed from citrate‐capped Au NPs by surface modification with semi‐fluorinated oligo(ethylene glycol) ligands (SFLs) in tetrahydrofuran (THF). However, the detailed formation mechanism has remained unclear.^[^ ^41^, ^42^, ^43^ ^]^ In this study, we revealed that NCs are formed through solute‐induced liquid–liquid phase separation, with interface stabilization by amphiphilic oligo(ethylene glycol) (OEG)‐modified NPs (**Scheme** **1**a,b). This method, which utilizes solute‐induced phase separation, provides nanodroplets suitable for NC formation and enables the formation of highly stable inorganic NCs through interfacial nanoparticle assembly, which cannot be achieved using conventional Pickering emulsions. Based on the formation mechanism, a simple and versatile method was developed to enable stable NC formation. Furthermore, by leveraging the formation mechanism in which water is extracted into the organic phase and salts become enriched, various materials—including molecules and nanoparticles—can be efficiently encapsulated through simultaneous enrichment (Scheme 1c). Material encapsulation in NCs consisting of bio‐adaptive and non‐reactive OEG‐NPs holds great potential for various applications, including DDSs and nanoreactors. To establish a versatile NC formation method, NC formation and evaluation were conducted under various conditions using OEG‐modified NPs. Importantly, our method demonstrated effective material encapsulation by leveraging the enrichment process in both NP and molecule encapsulation experiments.

## 2 Results and Discussion

### 2.1 Mechanism of NC Formation

First, the formation mechanism of NCs was investigated. In our previous study, Au NCs spontaneously formed when aqueous dispersions of citrate‐capped Au NPs were added to THF containing SFLs (Figure S2a, Supporting Information) and subsequently surface‐modified.^[^ ^41^ ^]^ Here, we focused on citrates presented in the citrate‐capped Au NP dispersion. Organic solvents such as THF are miscible with water, but when kosmotropic salt is dissolved in the water, the concentrated solute induces liquid–liquid phase separation. Recently, Wang et al. demonstrated that the spontaneous generation of nanodroplets by solute‐induced phase separation using kosmotropic salts and subsequent exploitation of the interface is useful for hollow NP formation.^[^ ^44^ ^]^ This means that emulsions can be formed even with miscible solvents by utilizing salt‐induced phase separation. Our previous system also contained citrate as a salt in a mixture of water and THF, and a similar phase separation is expected to occur. Therefore, the formation mechanism shown in Scheme 1a, b was predicted. Experimental results confirmed that a high concentration of aqueous sodium citrate solution and THF underwent phase separation, during which SFL‐Au NPs were observed to assemble at the interface (Figure S3a, Supporting Information). This interfacial assembly of NPs was not exclusive to SFL‐Au NPs but was also observed for Au NPs modified with EG6C11‐SH (Figure S3b, Supporting Information), a commercially available OEG ligand, suggesting the importance of amphiphilic OEG modification. Therefore, NC formation was performed via the self‐assembly of OEG‐Au NPs at the liquid–liquid phase‐separation interface induced by citrate. The formation of NCs using miscible solvents and the solute‐induced liquid–liquid interface is expected to allow the formation of nanoscale‐stable NCs more easily than conventional Pickering emulsions, as suggested by the formation mechanism. Furthermore, the solute enrichment process during NC formation is expected to be useful for effective material encapsulation (Scheme 1c).

### 2.2 Formation of Au NCs

**Figure 1:**
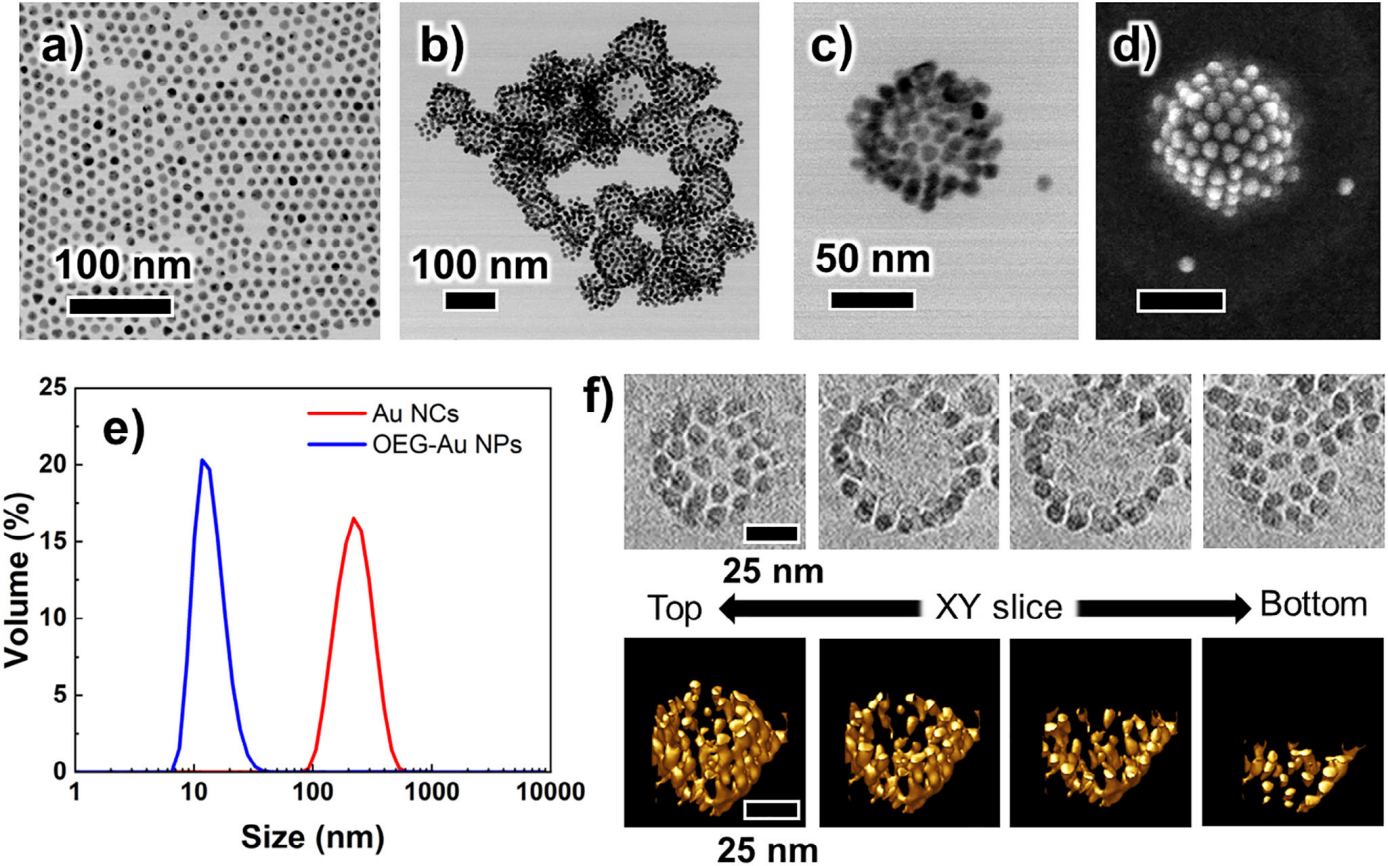
TEM images of a) OEG‐Au NPs and b, c) Au NCs. d) SEM image of (c). e) DLS results for OEG‐Au NPs dispersed in THF (blue line) and Au NCs in THF (red line). f) Reconstructed images (XY plane) and 3D models of Au NCs in dioxane from cryo‐ET tilt series. The scale bar in the upper left TEM image in (f) corresponds to 25 nm and is common to all TEM images. Note that the elliptical rather than spherical shape of the reconstructed NP model is due to the angular limitation (± 60°) in ET.

First, citrate‐capped Au NPs were modified with OEG‐ligands (Figure S4, Supporting Information), followed by NC formation according to the procedure illustrated in Scheme 1a. **Figure** **1**a–e shows the results of scanning transmission electron microscopic (STEM) observation and dynamic Light Scattering (DLS) of Au NCs formed from OEG‐Au NPs. TEM observation confirmed the successful formation of Au NCs. The size of the Au NCs was estimated to be ≈100 nm, which is in good agreement with the DLS results indicating a relatively narrow size distribution (polydispersity index (PDI) = 0.13). In addition, extinction spectra showed a redshift of the plasmonic peak from 523 to 546 nm, suggesting plasmon coupling due to NP assembly (Figure S5, Supporting Information). On the other hand, when pure water was used instead of a citrate solution, although the formation of aggregates was confirmed through DLS and extinction spectra, NC formation was not confirmed by TEM observation (Figure S6, Supporting Information). This suggests the occurrence of simple aggregate formation or the formation of unstable NCs, indicating that the presence of citrate, at least, is essential for the formation of stable NCs. Au NCs were successfully formed from Au NPs modified with OEG ligands without the need for specific fluorinated ligands. This suggests that amphiphilic OEG domains play a crucial role in stabilizing the THF/aqueous citric acid solution interface required for Au NC formation. Au NCs were then formed in 1,4‐dioxane, which has solvent parameters similar to those of THF. Au NCs were also stably formed in dioxane without significant size change (Figure S7, Supporting Information). This suggested that NCs could be formed in not only THF but also other solvents that provide similar liquid–liquid interfaces. Furthermore, cryo‐TEM and cryo‐electron tomographic (ET) observations were performed to confirm the assembled structures as the structures can be affected by the drying process in STEM sample preparation. It should be noted that cryo‐TEM imaging in organic solvents has seldom been reported. Figure 1f shows *XY* slice images and 3D images of Au NCs in dioxane reconstructed from cryo‐ET tilt series. The slice TEM images of Au NCs indicated that the obtained structures were hollow 3D capsules. The corresponding 3D model also shows a hollow capsule structure. The tilt and the *XY* slice movies of the reconstructed images are provided as Movies S1 and S2 (Supporting Information), respectively. These results show that most Au NPs formed hollow 3D capsule structures. In addition, the NC structure observed by cryo‐TEM is almost the same as that observed by STEM, indicating that the NC structure is highly stable and undergoes negligible change during the drying process. The above results demonstrated that uniform and stable NCs can be obtained from OEG‐Au NPs via self‐assembly at a solute‐induced liquid–liquid interface. The degree of citrate concentration was calculated based on the observed NC structure (See Figure S1, Supporting Information). Assuming an NC size of 100 nm, each capsule consists of ≈190 NPs, with an internal space of 2.7 × 10^5^ nm^3^. The number of NCs can be determined based on the NP concentration, and the total internal volume sums up to 4.0 × 10^−3^ µL. The amount of citric acid solution added for NC formation was 10 µL, indicating that water extraction led to a volume reduction by a factor of ≈2500. This suggests that this method has the potential to encapsulate water‐soluble molecules and water‐dispersible particles while achieving a more than 2000‐fold enrichment through water extraction. The citrate concentration becomes ≈1.8 m, which is high and close to the saturation concentration, supporting our hypothesized NC formation mechanism. Wang et al. synthesized hollow NPs of ≈100 nm via solute‐induced phase separation, which initiates the nucleation of droplets from a homogeneous solution.^[^ ^44^ ^]^ In this study, however, solute enrichment occurs inside the NCs because the droplets are made smaller by water extraction. Therefore, this method is also expected to effectively encapsulate materials through 2500‐fold enrichment.

### 2.3 Reversible Formation of Au NCs via Solvent Change

**Figure 2:**
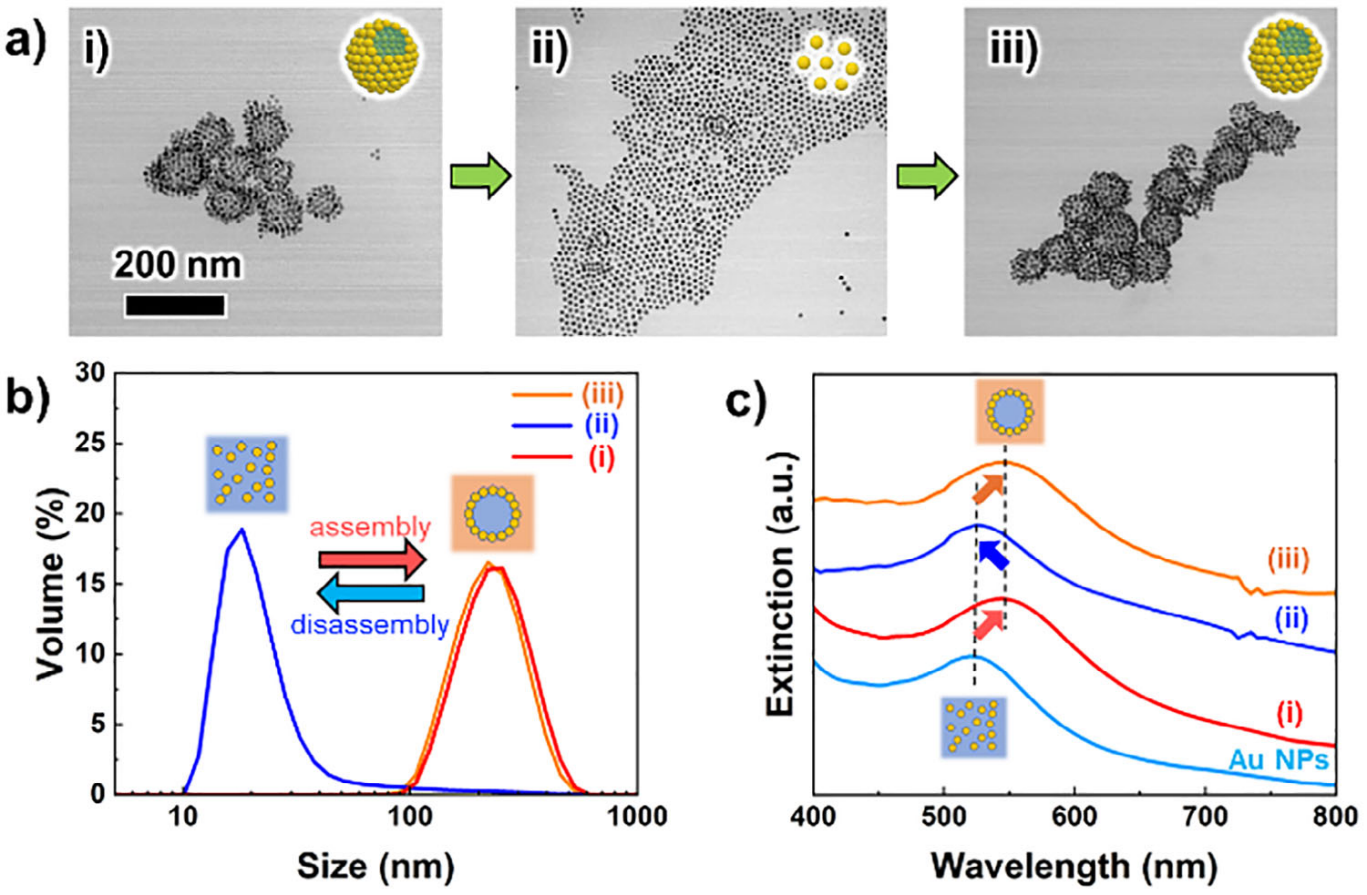
a) TEM images of (i) Au NCs in THF, (ii) disassembled Au NPs in water, and (iii) reformed Au NCs in THF. The scale bar in (i) corresponds to 200 nm and is common to all TEM images. b) DLS results of (i–iii). (c) Extinction spectra of (i–iii) and aqueous dispersion of OEG‐Au NPs.

Next, we undertook the reversible formation of Au NCs. NC disassembly and reformation are crucial for efficient drug release in DDSs and NP recycling in nanoreactors. Our previous results showed that Au NCs formed from SFL‐Au NPs have stable and robust NC structures due to strong interactions between SFLs (Figure S3, Supporting Information), retaining their structure even in water despite the formation mechanism being based on Pickering emulsion.^[^ ^42^ ^]^ In contrast, Au NCs formed from OEG‐Au NPs underwent disassembly in water. Therefore, the reversible formation of NCs using OEG‐Au NPs was evaluated. **Figure** **2** shows TEM images, DLS results, and extinction spectra during the repeat formation of Au NCs. The results showed that Au NCs were disassembled and dispersed as individual Au NPs in water, demonstrating that the robustness of NCs can be adjusted by modifying the design of the OEG ligands. As both the flexibility and robustness of structure are crucial for the retention and release of encapsulated materials, the ability to adjust structural robustness for specific purposes represents an additional advantage of this method. OEG‐Au NPs were then collected by centrifugation and Au NCs were re‐formed in THF in the same way as described above. Consequently, Au NCs could be re‐formed without significant change in size, demonstrating the repeated formation of Au NCs. DLS results and extinction spectra also support repeatable Au NC formation with good accuracy. Such reversible NC formation was achieved at least three times (Figure S8, Supporting Information). The Au NCs formed by this method are also highly reusable, which is advantageous to their various applications.

### 2.4 Effects of Solvent Quality and NP Concentration on NC Formation

**Figure 3:**
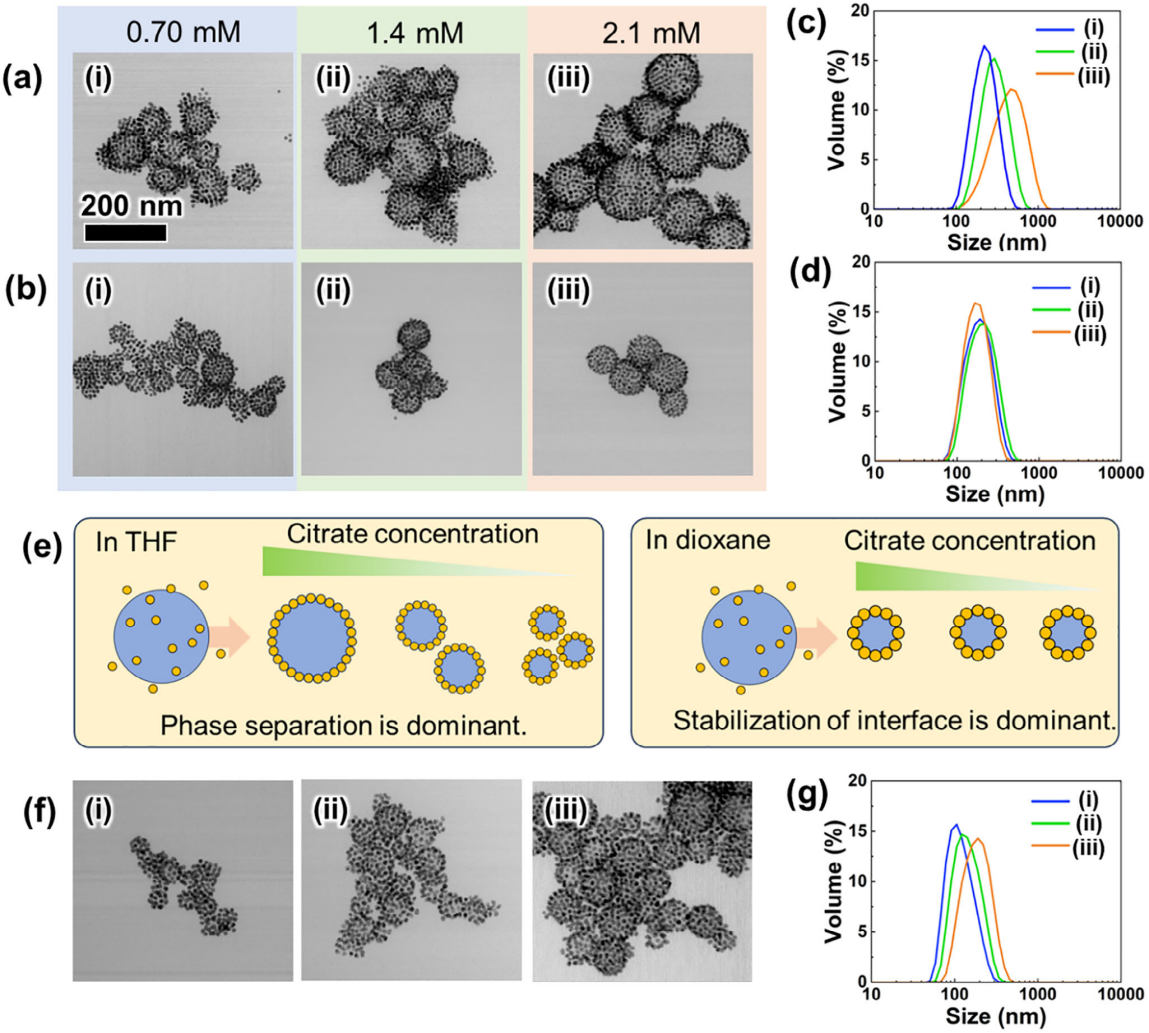
TEM images of Au NCs formed in a) THF and b) dioxane with different citrate concentrations, (i) 0.70 mM, (ii) 1.4 mM, and (ii) 2.1 mM. c,d) DLS results of Au NCs in (a) and (b). e) Proposed size determination process in THF and dioxane. f) TEM images of Au NCs formed in dioxane at different Au NP concentrations: (i) 19.2 nM, (ii) 9.6 nM, and (iii) 4.8 nM. The scale bar in (a‐i) corresponds to 200 nm and is common for all images. g) DLS results of Au NCs in (f).

Next, Au NCs were formed under various conditions to clarify the formation mechanism further and achieve controlled formation. Based on our formation mechanism, the size of the Au NCs can be controlled by changing the balance between the surface area of the water droplets and the number of particles. Thus, we first controlled the capsule size by varying the total volume of the water droplets after phase separation by changing the citrate concentration. OEG‐Au NPs were dispersed in aqueous citrate solutions of varying concentrations, followed by the formation of Au NCs. **Figure** **3**a,c shows the TEM images and DLS results of Au NCs formed in THF with different citrate concentrations from 0.70 to 2.1 mM. Au NCs were obtained at all concentrations, with the size of the Au NCs systemically increasing with increases in the salt concentration. When the concentration of citrate is increased, phase separation is expected to occur earlier, and the total volume of droplets should be increased. As the total number of Au NPs was constant, larger Au NCs with a lower specific surface area would therefore be formed. The increase in NC size observed in the TEM images is in good agreement with theoretical predictions (Figure S9, Supporting Information). The results demonstrated that the size of Au NCs can be easily controlled by changing the solvent condition, supporting the formation mechanism of Au NCs. Subsequently, the amount of citrate was fixed, and the water content was increased from 2 to 4 vol% to investigate the effect of water content on NC formation. The size of Au NCs was significantly increased to ≈400 nm when THF was used as a solvent (Figure S10a,c, Supporting Information). This suggested that the equilibrium of phase separation was significantly affected by changing the water content in THF. These results demonstrate that size‐controlled and stable Au NCs can be achieved by optimizing the solvent conditions. Interestingly, a different trend was observed for dioxane than for THF (Figure 3b,d), as the size of the Au NCs did not change significantly with variations in citrate concentrations. Additionally, the size of Au NCs didn't show a significant change in dioxane even when the water content was varied (Figure S10b,d, Supporting Information). This is consistent with our previous report,^[^ ^45^ ^]^ indicating that water content does not affect size within an appropriate range of conditions in dioxane. These results suggest that the dominant factors in NC size determination differ between THF and dioxane. To identify the causes of these differences, the phase separation behavior of the solvents was compared (Figure S11, Supporting Information). THF/citrate aqueous showed phase separation, whereas sodium citrate was gradually precipitated in dioxane, even after initial phase separation, showing that dioxane strongly extracted water and caused crystal precipitation rather than phase separation. In contrast, the liquid–liquid interface was stabilized with OEG‐Au NPs without citrate precipitation even after more than a week. This suggests that OEG‐Au NPs can induce phase separation via stabilization of the liquid–liquid interface even when dioxane is used as the solvent. Because of this difference in affinity for water, the size determination process differs between THF and dioxane. Phase separation is significantly influenced by salt concentration in THF, while interfacial stabilization is considered the dominant factor in size determination in dioxane, as water is strongly extracted into dioxane (Figure 3e). These results suggest that the use of THF and dioxane allows size control depending on the salt concentration and the formation of Au NCs of a certain size without any effect of the salt concentration, respectively. Furthermore, it should be noted that NC size could be controlled by changing the number of Au NPs, even when dioxane was used (Figure 3f,g). The results show a trend toward smaller NCs with higher NP concentrations. The observed correlation between particle concentration and NC size, as revealed by TEM analysis, resembles that of conventional Pickering emulsions, suggesting that precise size control can be achieved by tuning the NP concentration (Figure S12, Supporting Information). Thus, Au NCs of controlled size can be formed stably in dioxane with little influence from the solvent conditions.

The above results demonstrated that Au NCs can be stably formed at a citrate‐induced liquid–liquid interface. Citrate is a natural nutrient and a biocompatible molecule commonly found in food and other products, making it a promising candidate for applications in DDSs. Here, phosphate, a compound commonly used in biological applications such as buffers, was also employed to form NCs in a similar manner, demonstrating the applicability of this method with salts other than citrate. Au NC formation with phosphate was carried out in THF and dioxane (Figure S13, Supporting Information). When THF was used, no assembled structures were observed on TEM observations. However, the small round voids observed on TEM observations and DLS results imply the formation of small Au NCs. This may be attributed to the differing phase separation behavior of THF‐citrate and THF‐phosphate aqueous solutions (Figure S13e, Supporting Information). This indicates that NC formation is also affected by the solvent conditions in THF and optimization is required. In contrast, Au NCs were successfully formed in dioxane. This indicates that the formation of Au NCs is influenced less by the salt species, similar to the effect of salt concentration when dioxane is used as a solvent. These results suggested that Au NCs can be formed by using not only citrate but also other salt species. It also means that our method is expected to effectively encapsulate charged molecules while simultaneously concentrating them.

### 2.5 Formation of NCs from Fe_3_O_4_ NPs Dispersed in the Organic Solvent

**Figure 4:**
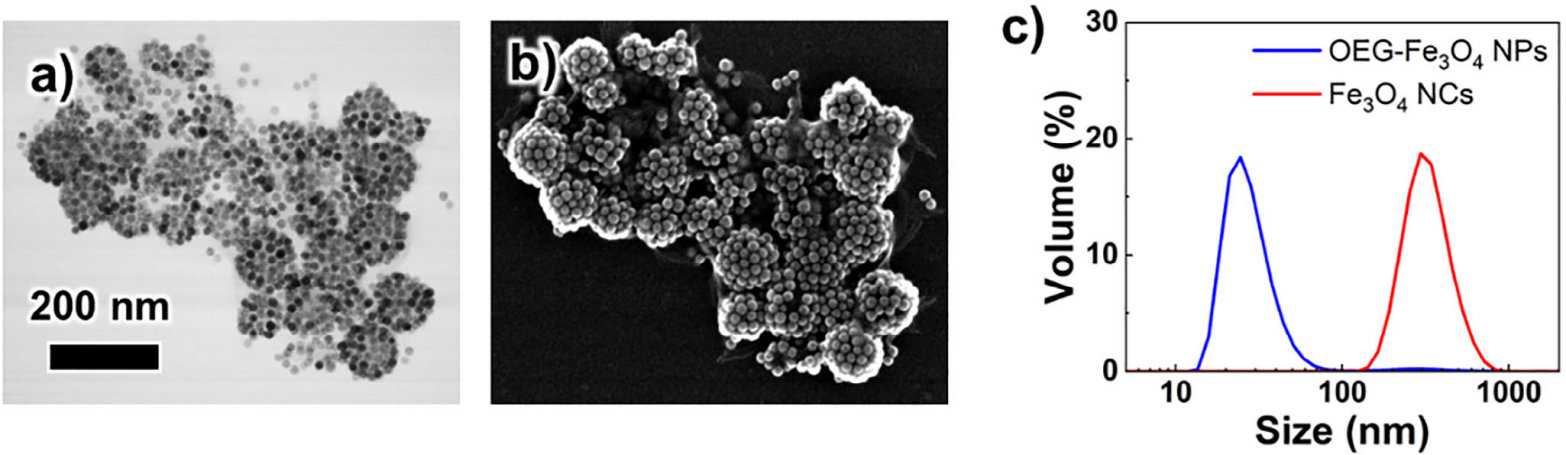
a) TEM and b) SEM images of Fe_3_O_4_ NCs formed in THF. The scale bar in (a) corresponds to 200 nm and is common for all images. c) DLS results of OEG‐Fe_3_O_4_ NPs and Fe_3_O_4_ NCs in THF.

Our method was then extended to magnetic Fe_3_O_4_ NPs dispersed in an organic solvent (Scheme 1b), demonstrating that NCs can be formed even when we use other inorganic NPs. Here, m‐EG8C12‐PO_3_H_2_ (Figure S2c, Supporting Information), a phosphonic acid ligand that binds strongly to metal oxide surfaces containing Fe_3_O_4_ NPs and is a structural analog of EG6C11‐SH, was used. Fe_3_O_4_ NPs were modified with OEG‐ligands (Figure S14, Supporting Information), followed by the formation of Fe_3_O_4_ NCs. **Figure** **4** shows the STEM images and DLS results of Fe_3_O_4_ NCs. STEM observations confirmed that Fe_3_O_4_ NCs were successfully formed from 20 nm Fe_3_O_4_ NPs with a uniform size of ≈100 nm. DLS results also confirmed the formation of the NP assembly with a relatively narrow size distribution (PDI = 0.22), which agrees with the STEM observations. These results suggest that the surface properties of OEG‐inorganic NPs play a crucial role in NC formation and that NC structures can form irrespective of the core NP type. In addition, Fe_3_O_4_ NCs were also formed when m‐EG9C2‐PO_3_H_2_ (Figure S2d, Supporting Information) with a shorter alkyl domain was used as the ligand (Figure S15, Supporting Information). This indicates that the terminal OEG domain is important for NC formation and that there is still room to modify the ligand design, as previously reported.^[^ ^41^, ^42^ ^]^ Next, we undertook the size control of Fe_3_O_4_ NCs. As mentioned above, NC size is affected by the balance between droplet volume and the number of NPs. Thus, NC size was controlled by varying the concentration of Fe_3_O_4_ NPs (Figure S16, Supporting Information). The results show that the size of the Fe_3_O_4_ NCs was systemically changed through changes in the concentration of Fe_3_O_4_ NPs as expected. These results demonstrated that NC structures with controlled size can be formed not only from Au NPs but also from other inorganic NPs by our method.

### 2.6 Encapsulation of Au NPs and DNA into NCs

**Figure 5:**
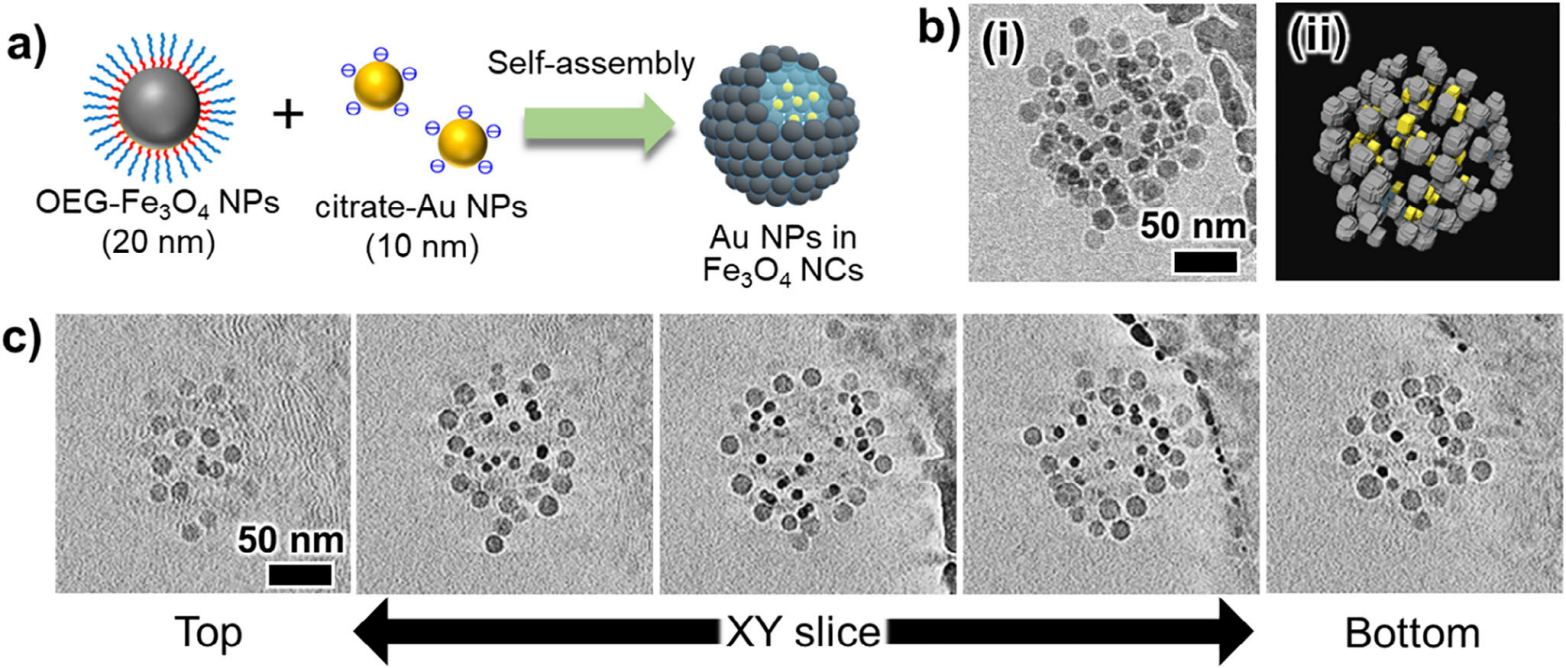
a) Schematic illustration of NP encapsulation. b) (i) Cryo‐TEM image of Fe_3_O_4_ NCs encapsulating 10 nm Au NPs in vitrified THF. (ii) 3D reconstruction model from cryo‐ET using Amira software. Fe₃O₄ NPs are shown in gray, while Au NPs are shown in yellow. Au NPs and Fe_3_O_4_ NPs were distinguishable by differences in particle size and electron density, with Au NPs appearing darker. c) Reconstructed slice images (XY plane) of Fe_3_O_4_ NCs encapsulating Au NPs. The scale bars in (b) and (c) correspond to 50 nm and are common for all images. The edge of the gold‐sputtered carbon film is visible in the upper right of some images.

We next demonstrated the enhanced encapsulation efficiency of NPs and molecules into inorganic NCs (Scheme 1c). First, Au NPs were encapsulated into Fe_3_O_4_ NCs in THF (**Figure** **5**a). Based on our formation mechanism, water‐soluble or dispersible materials are expected to be effectively encapsulated into NCs through the enrichment process. Therefore, water‐dispersible citrate‐capped Au NPs were encapsulated into the Fe_3_O_4_ NCs as a test target material. The cryo‐TEM images suggest that the outer layer (shell) of the formed NCs is composed of Fe_3_O_4_ NPs and the inner side is Au NPs based on the differences in particle diameter and electron density (Figure 5b (i)). The reconstructed 3D model and slice images (*XY* plane) from cryo‐TEM tomography demonstrated that Au NPs were successfully encapsulated in the Fe_3_O_4_ NCs (Figure 5b (ii), Figure 5c). The tilt and the *XY* slice movies of reconstructed images are provided as Movies S3 and S4 (Supporting Information). Additionally, energy dispersive X‐ray spectroscopy mapping confirms that Au NPs can be distinguished from Fe_3_O_4_ NPs based on differences in contrast and particle size (Figure S17, Supporting Information), further supporting the encapsulation of Au NPs. It is also worth noting that the encapsulated Au NPs remain dispersed without aggregation and can diffuse freely within the NCs. This is advantageous for applications such as nanoreactors encapsulating catalytic NPs.

**Figure 6:**
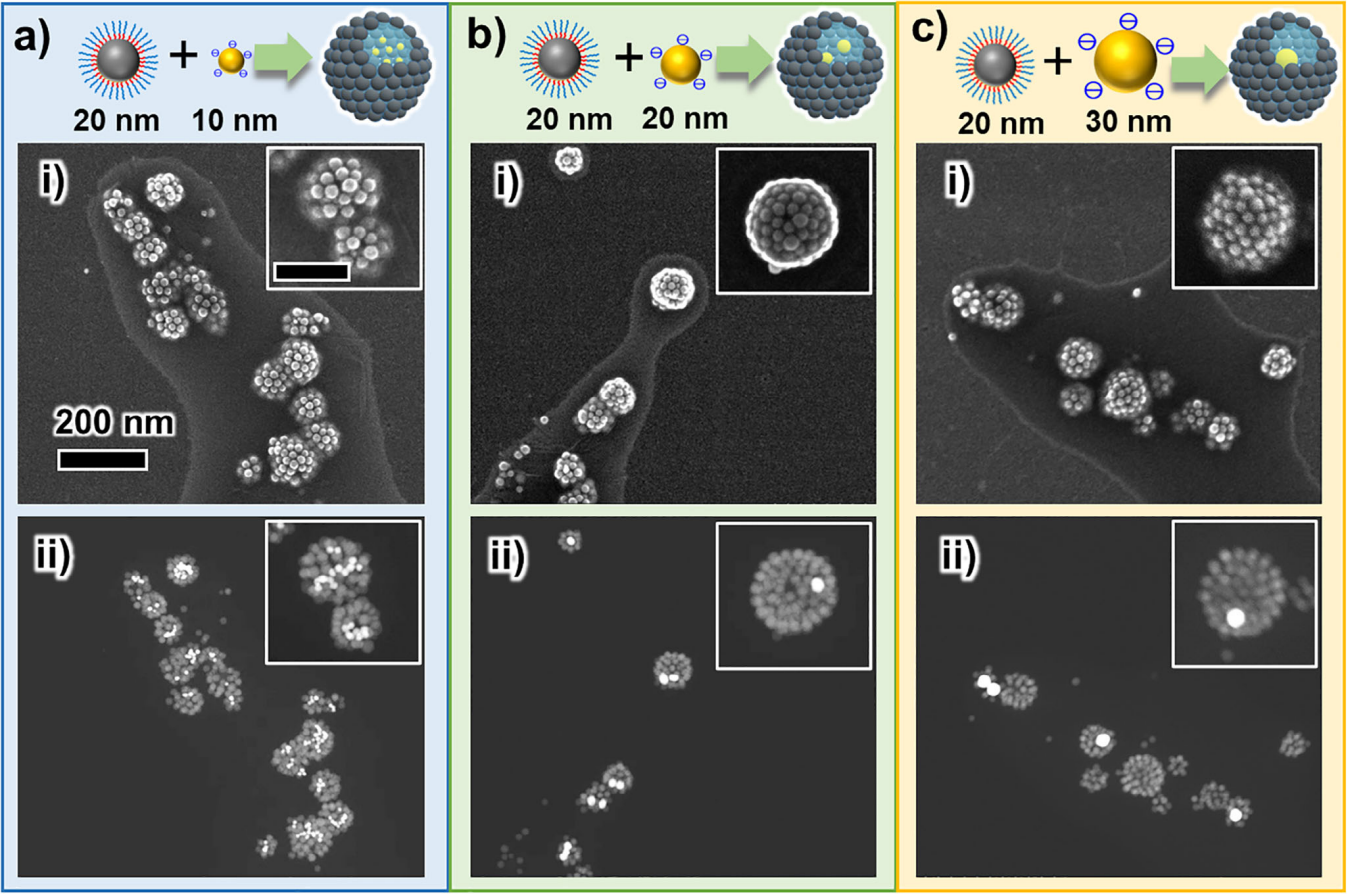
SEM and HAADF‐STEM images of Au NPs encapsulated Fe_3_O_4_ NCs structures formed from different sizes of Au NPs, a), 10 nm, b) 20 nm, and c) 30 nm. (i) and (ii) in (a–c) show SEM and HAADF‐STEM images, respectively. Au NPs are observed as bright white particles in HAADF‐STEM images due to their high electron density. The scale bars in (a‐i) and the insets of (a‐i) correspond to 200 nm and 100 nm, respectively, and are common for (a–c).

**Figure** **6** shows STEM images of Fe_3_O_4_ NCs encapsulating Au NPs (10–30 nm). From the SEM images, the formation of NCs was confirmed and the outer shells were formed only from 20 nm NPs, which appeared to be Fe_3_O_4_ NPs (Figure 6a–c (i)). Meanwhile, high‐angle annular dark‐field (HAADF)‐STEM observations confirmed the encapsulation of Au NPs due to the difference in electron density between Au and Fe_3_O_4_ (Figure 6a–c (ii)). These results show that Au NPs were successfully encapsulated into Fe_3_O_4_ NCs regardless of the particle size. NC formation and NP encapsulation were also confirmed from the extinction spectra and DLS results (Figure S18, Supporting Information). The number of NCs can be calculated similarly to the method used for Au NCs, and based on the concentration of Au NPs, it is estimated that 12, 1.5, and 0.41 particles are encapsulated per NC when using Au NPs of 10, 20, and 30 nm, respectively. These calculation results are consistent with the STEM observations, with no Au NPs observed outside of the Fe_3_O_4_ NCs, suggesting that almost all Au NPs were successfully encapsulated.

**Figure 7:**
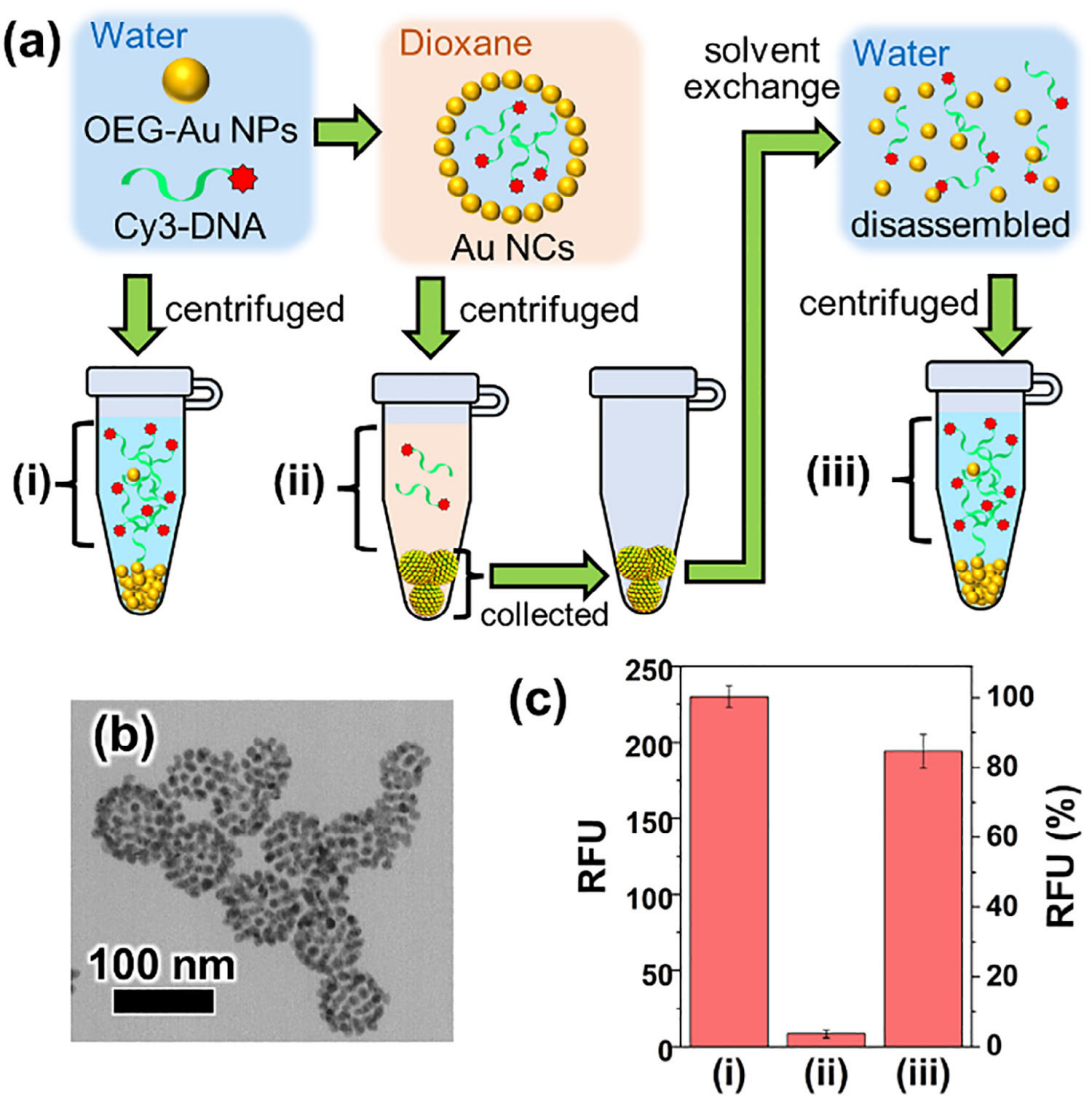
a) A schematic illustration of Cy3‐DNA encapsulation and fluorescence measurements to confirm successful encapsulation. b) TEM image of Au NCs encapsulating Cy3‐DNA. The scale bar corresponds to 100 nm. c) The result of RFU of fluorescence measurements of each state (i‐iii) in (a). The bar graph shows RFU at 565 nm and the error bars represent the standard deviation (n = 3).

Finally, molecular encapsulation was performed. Here, fluorescent dye‐modified DNA (Cy3‐DNA) was used as a test molecule. DNA, being a polyionic molecule, is expected to be encapsulated, with the fluorescent moiety facilitating its evaluation. **Figure** **7**a shows a schematic illustration of Cy3‐DNA encapsulation and its evaluation through fluorescence measurements. TEM observations show that Au NCs can be formed even when Cy3‐DNA is added (Figure 7b). Encapsulation was then confirmed by fluorescence measurements. For evaluation, (i) a simple mixture of Au NPs and Cy3‐DNA was centrifuged and the supernatant was measured first as an original DNA concentration, followed by measurements of (ii) the unencapsulated molecules and (iii) the encapsulated molecules. The results suggest that most of the Cy3‐DNA was encapsulated (Figure 7c). The sum of the relative fluorescent units (RFU) of (ii) and (iii) is smaller than that of (i), possibly due to the loss of NCs in the process of removing organic solvents after centrifugation or quenching of Cy3 by some Au NPs that did not precipitate. This result indicates that at least 80% of DNA was successfully encapsulated. As mentioned above, ≈2500‐fold enrichment occurs during NC formation, suggesting that this method can encapsulate molecules with over 2000‐fold enrichment, even accounting for minor losses. These results indicate that this method enables the enhanced encapsulation of a wide variety of materials, from NPs to molecules, although the encapsulated material must be less soluble or poorly dispersed in the organic solvent side. Effective molecular encapsulation can be a great advantage for applications as DDS carriers, especially in applications involving costly and rare drugs, such as DNA‐ and RNA‐based gene delivery. Notably, although our NCs tend to collapse in aqueous solutions, our previous reports have demonstrated that cross‐linking Au NPs on NCs can retain the NC structure even in water and prevent leakage of molecules, making it available for DDSs.^[^ ^42^ ^]^ By applying such a cross‐linking strategy to the present system, structural stability in aqueous environments could be significantly enhanced, and the method could be further developed for DDS applications, including gene delivery. Therefore, our method appears to be a promising technology for the formation of stable NCs that encapsulate the target material while simultaneously concentrating them.

## 3 Conclusion

In this study, we developed a versatile method for inorganic NC formation. Highly stable Au NCs were formed from OEG‐inorganic NPs via self‐assembly at solute‐induced THF or dioxane/citrate aqueous liquid–liquid interfaces. The size of the NCs could be controlled by varying conditions such as organic solvent, salt concentration, and salt type. Our calculations estimated that solutes were enriched by over 2000‐fold through water extraction, suggesting the potential for effective material encapsulation. Indeed, the encapsulation experiment with Cy3‐DNA indicates that molecules can be effectively encapsulated using this enrichment process. Our method was subsequently applied to magnetic Fe_3_O_4_ NPs to demonstrate that NCs can also be formed from other types of inorganic NPs. The results demonstrated that size‐controlled NCs can be obtained regardless of the NPs, indicating that this method is a versatile technique for fabricating various inorganic NCs from NPs modified with simple OEG ligands. Finally, the encapsulation of Au NPs into Fe_3_O_4_ NCs was carried out to visually demonstrate that our method allows us to encapsulate materials into inorganic NCs through NC formation. Most Au NPs were encapsulated into Fe_3_O_4_ NCs through the enrichment process, indicating that this method is useful for effective material encapsulation. These results demonstrate that this method enables the formation of size‐controlled, stable inorganic NCs while effectively encapsulating substances into the NCs. This method holds promise for applications in DDSs, including gene delivery, by effectively encapsulating drugs in NCs composed of biocompatible OEG‐NPs. Effective encapsulation of molecules and NPs also facilitates the development of nanoreactors that leverage catalytic NPs^[^ ^46^, ^47^, ^48^, ^49^, ^50^ ^]^ or localized heating phenomena such as plasmon heating^[^ ^51^, ^52^ ^]^ and magnetic induction heating.^[^ ^53^, ^54^, ^55^, ^56^ ^]^ In addition, the interior of the plasmonic NCs serves as a hot spot, enabling reaction monitoring via SERS.^[^ ^18^, ^57^, ^58^ ^]^ The formation of hierarchical NC structures composed of different particles enables the fabrication of metamaterials^[^ ^59^, ^60^ ^]^ through precise control of interparticle interactions. Thus, this versatile technology for forming NCs from various inorganic NPs and encapsulating materials within them opens new avenues for advanced applications.

## Conflict of Interest

The authors declare no conflict of interest.

## Supporting information



Supporting Information

Supplemental Movie 1

Supplemental Movie 2

Supplemental Movie 3

Supplemental Movie 4

## Data Availability

The data that support the findings of this study are available in the supplementary material of this article.
