# Supplementary material for: Versatile Nanoparticle Capsule Formation With Enhanced Encapsulation Efficiency via Solute‐Induced Liquid–Liquid Phase Separation
Source: Small. 2025 May 7;21(30):2502573. doi: 10.1002/smll.202502573 (PMC12306390; doi:10.1002/smll.202502573)
Supplement: Supplementary file 1 — Supporting Information [file SMLL-21-2502573-s002.pdf]

NANO · MICRO
small

Supporting Information

for *Small*, DOI 10.1002/smll.202502573

# Versatile Nanoparticle Capsule Formation With Enhanced Encapsulation Efficiency via Solute-Induced Liquid–Liquid Phase Separation

*Takehiro Yachi, Honoka Watanabe, Rumi Niwa, Daisuke Unabara, Tasuku Hamaguchi, Yusuke Yonamine, Koji Yonekura, Kuniharu Ijiro* and Hideyuki Mitomo**

*Supporting Information*

# Versatile Nanoparticle Capsule Formation with Enhanced Encapsulation Efficiency via Solute-Induced Liquid-Liquid Phase Separation

Takehiro Yachi[1], Honoka Watanabe[2], Rumi Niwa[3,4], Daisuke Unabara[4], Tasuku Hamaguchi[3,4], Yusuke Yonamine[1], Koji Yonekura[3,4,5], Kuniharu Ijiro*[1], and Hideyuki Mitomo*[1,4]

[1] Research Institute for Electronic Science, Hokkaido University, Sapporo, 001-0020, Japan

[2] Graduate School of Life Science, Hokkaido University, Sapporo, 060-0810, Japan

[3] Graduate School of Life Science, Tohoku University, Sendai, 980-8577, Japan

[4] Institute of Multidisciplinary Research for Advanced Materials, Tohoku University, Sendai, 980-8577, Japan

[5] Biostructural Mechanism Laboratory, RIKEN SPring-8 Center, Sayo, Hyogo 679-5148, Japan

# Experimental

## Materials & Instruments

Stabilizer-free tetrahydrofuran (THF), 1,4-dioxane, and sodium citrate anhydrous were purchased from FUJIFILM Wako Pure Chemical Corporation (Japan). 11-mercaptoundecanol hexamethyleneglycol ether (**EG6C11-SH**) (**Figure S2b**) was purchased from Dojindo Laboratories (Japan). Cy3-DNA (a 15-base DNA strand modified with a Cy3 fluorescent dye at the 5′ end; sequence: 5′-[Cy3]-ACGAACGCCATGGGA-3′) was purchased from Eurofins Genomics (Germany). Citrate-capped Au NPs (10 nm, 20 nm, and 30 nm) were purchased from BBI Solutions (UK). Twenty nm $Fe_3O_4$ NPs were purchased from Cytodiagnostics Inc. (Canada). (2,5,8,11,14,17,20,23,26-nonaoxaoctacosan-28-yl) phosphonic acid (**m-EG8C12-PO$_3$H$_2$**) (**Figure S2c**) and (2,5,8,11,14,17,20,23-octaoxapentatriacontan-35-yl) phosphonic acid (**m-EG9C2-PO$_3$H$_2$**) (**Figure S2d**) were purchased from BroadPharm (USA). Scanning transmission electron microscopy (STEM) observations were carried out using a STEM HD-2000 system (Hitachi High-Tech Manufacturing and Service Co., Ltd., Japan) with 200 kV acceleration voltage. Extinction spectra were measured using a V-730 or a V-770 UV–vis spectrometer (JASCO Corporation, Japan). Dynamic light scattering (DLS) measurements were performed using a Zetasizer nano ZS (Malvern Panalytical Ltd, UK). Cryo-TEM observations were performed using a CRYO ARM™ 300II system (JEOL, Japan) operating at an acceleration voltage of 300 kV. Fluorescence measurements were performed using a NanoDrop 3300 Fluorospectrometer (Thermo Fisher Scientific, USA).

## Methods

**Formation of Au NCs.** The representative procedure was as follows. As the first step, surface modification of Au NPs was carried out. A 500 μL water dispersion of Au NPs (10 nm, BBI Solutions) was centrifuged at 20,000 *g* for 30 min. Then, 50 μL of 2 mM EG6C11-SH in water and 450 μL of water was added and the mixture was stirred overnight. The resulting dispersion was centrifuged at 14,100 *g* for 1h and washed with water 3 times to remove excess ligands. Subsequently, Au NPs were dispersed in 10 μL of 0.70 mM sodium citrate and added into 490 μL THF (the final concentration of Au NPs was 9.5 nM). The mixture was then quickly mixed using a vortex mixer for 3 min. Next, the resulting dispersion was let stand overnight, and Au NCs were finally obtained. The formation of Au NCs was also carried out using 1,4-dioxane instead of THF to investigate the effect of organic solvent.

**Reversible formation of Au NCs.** Reformation of Au NCs was carried out as follows. Au NCs formed from EG6C11-SH-modified Au NPs were sonicated in water and dispersed into individual NPs. The Au NPs were recovered by centrifugation at 20,000 *g* for 30 min, and then dispersed in

10 μL of 0.70 mM sodium citrate aqueous solution. Au NCs were formed in THF in the same manner as the representative procedure.

**Size control of Au NCs with different conditions.** The size of Au NCs was controlled by changing the citrate concentration or water content. The citrate concentration was set at 0.70 mM, 1.4 mM, and 2.1 mM, and Au NCs were formed under the same conditions as used for the representative Au NCs formation to investigate the effect of salt concentration. Au NCs were also formed at different water contents. The total amount of citrate was fixed to be the same as in the case of 0.70 mM, while the water content was increased from 2 vol% to 10 vol%. For example, at 4%, OEG-Au NPs were dispersed in 20 μL of 0.35 mM sodium citrate and added to 480 μL of THF or dioxane to form Au NCs. Au NCs were also formed at different NP concentrations. The NP concentration was set at 4.8, 9.6, and 19.2 nM, and Au NCs were formed in dioxane following the same procedure as that used for representative Au NC formation to investigate the effect of NP concentration.

**Au NC formation with phosphate.** Au NCs were then formed with sodium phosphate instead of sodium citrate. OEG-Au NPs were dispersed in 10 μL of 0.70 mM sodium citrate aqueous solution and added to 490 μL of THF or dioxane. The resulting dispersion was then allowed to stand overnight to obtain Au NCs. Here, the concentration of Au NPs was half that used with citric acid.

**Formation of $Fe_3O_4$ NCs.** The representative procedure is shown below. As the first step, surface modification of $Fe_3O_4$ NPs was carried out. Fifty μL of a 5 mg/mL toluene dispersion of oleic acid-modified $Fe_3O_4$ NPs (20 nm, Cytodiagnostics Inc.) were centrifugated with 950 μL of ethanol at 14,100 *g* for 10 min. Next, 100 μL of chloroform and 900 μL of ethanol were added and centrifuged twice to remove excess oleic acid. Then, 250 μL of 2 mM m-EG8C12-$PO_3H_2$ or m-EG9C2-$PO_3H_2$ in $CHCl_3$ were added and mixed. After standing overnight, the resulting dispersion was washed with $CHCl_3$/hexane (1:3) mixture 3 times at 14,100 *g* for 10 min. Then, 490 μL of an OEG-$Fe_3O_4$ NPs dispersion in THF was prepared (the final concentration of $Fe_3O_4$ NPs was 10 μg/mL, 0.77 nM). Subsequently, 10 μL of 0.70 mM sodium citrate was added and mixed for 3 min. The resulting dispersion was allowed to stand overnight to obtain $Fe_3O_4$ NCs.

**Encapsulation of Au NPs into $Fe_3O_4$ NCs.** Citrate-capped Au NPs were encapsulated into the $Fe_3O_4$ NCs as a target material. Here, 10 nm, 20 nm, and 30 nm Au NPs were used. Firstly, citrate-capped Au NPs dispersion was centrifugated at 20,000 *g* for 20 min. The supernatant was removed and replaced with 0.70 mM sodium citrate aqueous solution. After repeating the above procedure 2 times, we obtained a Au NP dispersion in 0.70 mM sodium citrate aqueous solution. In this step,

the concentration of the Au NPs was adjusted to 10 nm:19.2 nM, 20 nm:2.4 nM, and 30 nm:0.68 nM, respectively. Then, 10 µL of the Au NPs dispersion was added into an OEG-$Fe_3O_4$ NPs dispersion in 490 µL THF (the final concentration of $Fe_3O_4$ NPs was 10 µg/mL, 0.77 nM). The resulting mixture was mixed for 3 min and stood for 12 h. The resulting dispersion was centrifugated at 1,000 *g* for 3 min and redispersed in 500 µg/mL THF.

**Encapsulation of fluorescent dye-modified DNA into Au NCs.** Cy3-DNA, a fluorescent dye-modified DNA, was encapsulated into Au NCs as a model molecule for molecular encapsulation. OEG-Au NPs were dispersed in 10 µL of 0.70 mM sodium citrate aqueous solution. Then, 10 µL of 5 µM Cy3-DNA in pure water was added, and the mixture was subsequently introduced - 4 - into 480 µL of dioxane (the final concentration of Cy3-DNA was 100 nM). The resulting mixture was rapidly mixed and left to stand overnight to obtain DNA-encapsulated Au NCs. Molecular encapsulation was evaluated by fluorescence measurement. After NC formation, the Au NCs were collected by centrifugation (2,000 *g*, 5 min) and the supernatant was analyzed to evaluate the amount of unencapsulated DNA. The Au NCs were then disassembled by dispersing them in water, and the encapsulated DNA and Au NPs were separated by centrifugation (14,100 *g*, 10 min). The fluorescence intensity of the supernatant was measured and compared with that of 100 nM Cy3-DNA solution to evaluate the internalization efficiency.

**Cryo-TEM tomography.** Sample preparation was carried out as follows. The NC dispersion in THF and 1,4-dioxane was deposited onto a glow discharged holey carbon TEM grid (Quantifoil R0.6/1 Cu 200 mesh, Quantifoil Micro Tools GmbH, Germany) that had been pre-coated with sputtered Au. Excess solvent was removed using filter paper in a semi-automated vitrification plunger, EM GP2 (Leica, Germany). The sample was then rapidly vitrified in liquid $N_2$. Cryo-TEM measurements were conducted at temperatures below 100 K using a CRYO ARM 300II system (JEOL, Japan) operated at an acceleration voltage of 300 kV with a nominal magnification of ×25,000. Dose-fractionated tilt images were acquired using SerialEM[1] and a custom-modified version of the FastTomo script.[2] A bi-directional tilt scheme was applied, covering an angular range from -60° to +60°, with 3° increments for Au NCs and 5° increments for $Fe_3O_4$ NCs with 10 nm Au NPs. The electron dose per each tilt angle was set to 0.6 $e^-/Å^2$ for Au NCs and 1.3 $e^-/Å^2$ for $Fe_3O_4$ NCs. Image acquisition was performed using a K3 direct electron detector (Gatan, AMETEK) in CDS and counting mode, respectively. Three-dimensional reconstructions of each the tilt series were carried out using the eTomo module within the IMOD software package.[3] 3D visualization of $Fe_3O_4$ NCs encapsulating Au NPs was conducted using Amira software (Thermo Fisher Scientific, USA).

**Calculation of concentration efficiency during NC formation.** Concentration efficiency was calculated from the following equations:

$$R_{drop} = R_{NC} - R_{NP}$$

$$n_{NPs} = S_{drop}/s_{NP} \times 0.9$$

$$V_{inner} = V_{drop} - (V_{NP} \times \frac{n_{NPs}}{2})$$

$$N_{NCs} = N_{NPs}/n_{NPs}$$

$$V_{total} = N_{NCs} \times V$$

$R_{NP}$: radius of NP, $R_{NC}$: radius of NC, $R_{drop}$: radius of droplet
$n_{NPs}$: number of NPs on a NC, $S_{drop}$: surface area of a droplet, $s_{NP}$: cross-sectional area of NP,
$N_{NCs}$: number of NCs, $N_{NPs}$: number of NPs
$V_{inner}$: inner space volume of a NC, $V_{NP}$: volume of a NP, $V_{drop}$: volume of a droplet,
$V_{total}$: total volume of inner space, $N_{NCs}$: number of NCs,

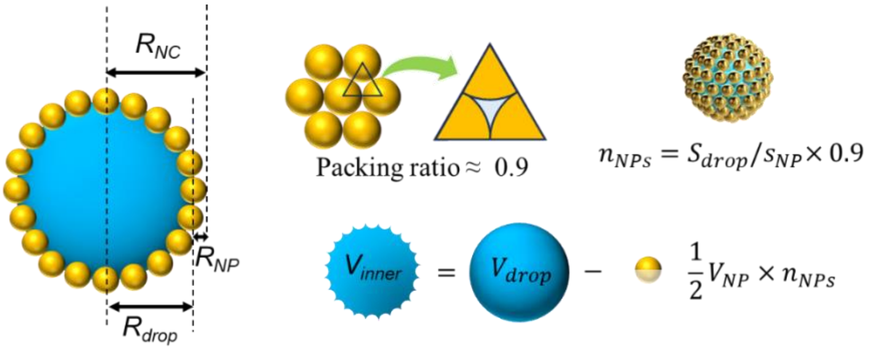


**Figure S1.** Definition of parameters in the calculation.

Here, the sizes of Au NPs and Au NCs are assumed to be 10 nm and 100 nm, respectively, with a ligand layer thickness of 1 nm on the particle surface. In addition, the surface coverage of NPs at the interface was assumed to be 0.9, representing the maximum packing ratio for circles. Based on these parameters, the inner volume of NC was calculated to be $2.7 \times 10^5$ nm$^3$, with a total number of NCs estimated at $1.5 \times 10^{10}$, resulting in a total inner volume of $4.0 \times 10^{-3}$ μL. The citric acid solution used was 10 μL, and the citric acid concentration is estimated to have increased approximately 2500-fold. The citric acid concentration, in this case, is 1.8 M, a high level close to saturation, which likely drives the phase separation and NC formation proposed in our assumption.

**Calculation of the number of Au NPs encapsulated in $Fe_3O_4$ NCs.** Assuming the sizes of $Fe_3O_4$ NPs and $Fe_3O_4$ NCs to be 20 and 100 nm, respectively, the total number of $Fe_3O_4$ NCs was calculated to be $9.9 \times 10^9$, which is similar to the number of Au NCs. The number of 10 nm, 20 nm, and 30 nm Au NPs was $1.2 \times 10^{11}$, $1.4 \times 10^{10}$, and $4.1 \times 10^9$, respectively, resulting in calculated encapsulation rates of 12, 1.5, and 0.41 particles per NC, respectively. This result is consistent with STEM observations, indicating that most Au NPs could be encapsulated through the concentration process.

## Supplementary Figures

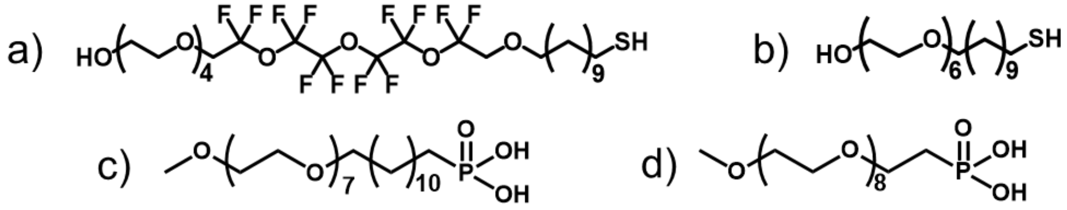


**Figure S2.** Chemical structures of (a) SFL, (b) $EG_6C_{11}$-SH, (c) m-$EG_8C_{12}$-$PO_3H_2$, and (d) m-$EG_9C_2$-$PO_3H_2$.

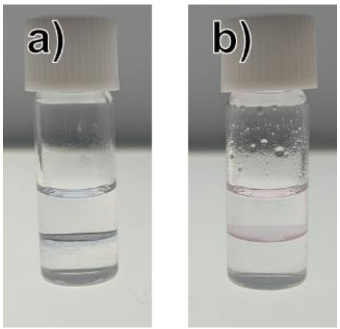


**Figure S3.** (a) SFL- and (b) EG6C11-SH-Au NPs assembled at the THF/citrate aqueous interface. The upper phase is THF and the lower phase is saturated sodium citrate solution. Interfacial coloration shows the assembly of Au NPs.

**Figure S3** shows pictures of SFL- and EG6C11-SH-modified Au NPs assembled at the THF/saturated citrate aqueous interface. SFL-Au NPs exhibited a blue-purple color at the interface due to plasmon coupling, resulting from aggregation driven by the strong interaction between SFLs. This result shows that amphiphilic OEG-Au NPs can stabilize such a liquid-liquid interface. Such interface stabilization is expected to contribute to the formation of stable Pickering emulsions.

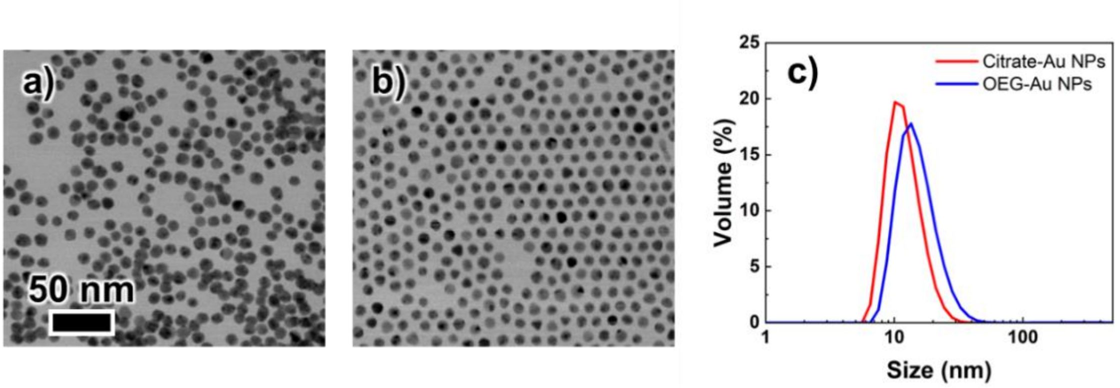


**Figure S4.** TEM images of Au NPs (a) before and (b) after OEG-ligand modification. (c) DLS result corresponding to (a) and (b).

**Figure S4** presents TEM images taken before and after surface modification. No significant change in particle size or shape was observed, whereas interparticle distances expanded, suggesting that successful surface modification. A slight increase in hydrodynamic diameter confirmed by DLS measurements further supports the progress of surface modification. The zeta potential also changed from -29 mV to -12 mV, further indicating that surface modification was achieved.

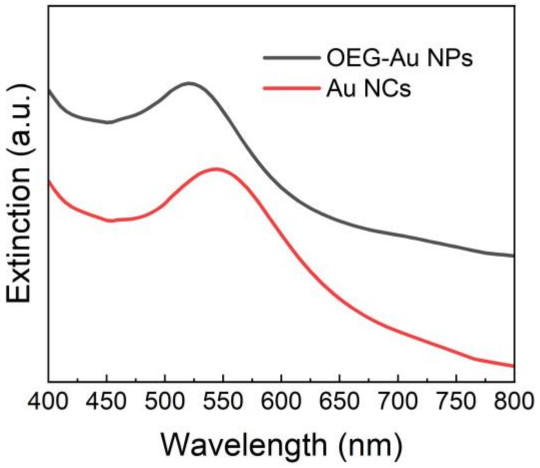


**Figure S5.** Extinction spectra before and after NC formation.

**Figure S5** shows the extinction spectra of OEG-Au NPs and Au NCs. The peak top shifted from 523 nm to 546 nm due to plasmon coupling, indicating that OEG-Au NPs formed assembled structures. This result also supports the formation of Au NCs.

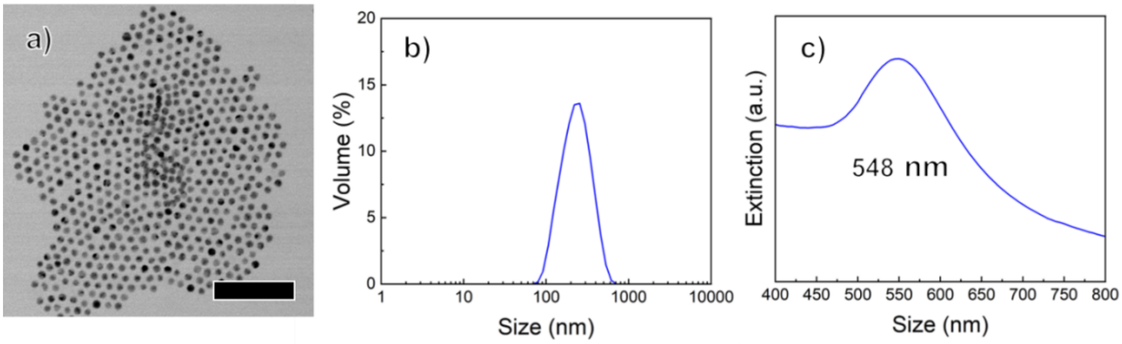


**Figure S6.** (a) TEM image, (b) DLS result, and (c) extinction spectra of OEG-Au NPs assembled with water instead of citrate aqueous solution.

**Figure S6** shows TEM image, DLS result, and extinction spectra when pure water was used for NC formation instead of 0.70 mM sodium citrate aqueous solution. Although aggregations were confirmed by DLS and peak shift in extinction spectra, the formation of NCs was not observed by TEM observation. This may be due to the aggregation of hydrated OEG-Au NPs in THF or the formation of less stable NCs. In any case, it is suggested that the presence of citrate is important for stable NC formation.

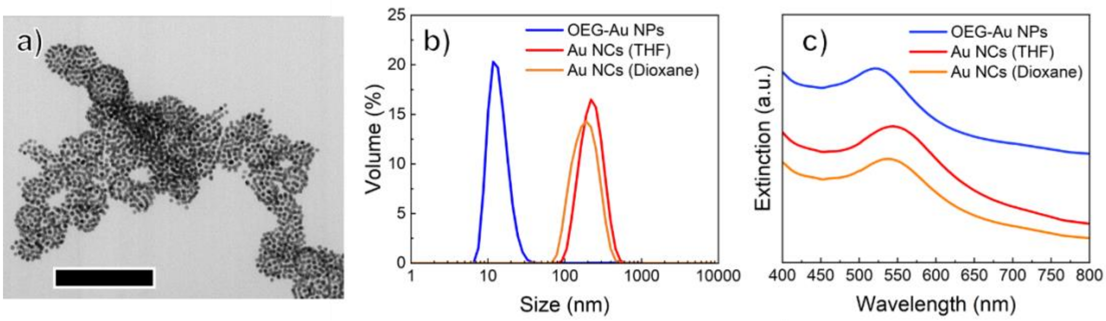


**Figure S7.** (a)TEM image of Au NCs formed in dioxane. The scale corresponds to 200 nm. (b) DLS results and (c) extinction spectra of OEG-Au NPs and Au NCs formed in THF and dioxane.

**Figure S7a** shows a TEM image of Au NCs formed in dioxane. Formation of Au NCs was also observed in dioxane with no significant change in size. **Figures S7a,b** show DLS results and extinction spectra of Au NCs formed in THF and dioxane, respectively. Similar results were obtained for either THF or dioxane solvent, indicating that similar NC structures can be obtained.

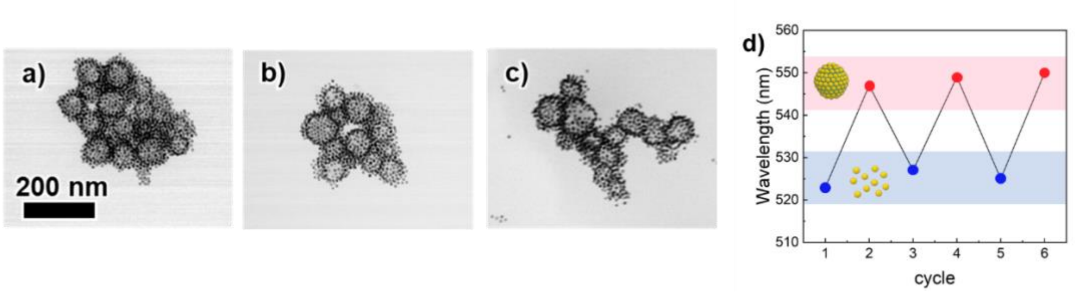


**Figure S8.** TEM images of Au NCs obtained through repeated formations: (a) first, (b) second, and (c) third cycles. (d) Plasmon peak positions during the assembly-disassembly cycles.

**Figure S8** demonstrates the repeatability of NC formation. TEM images reveal only slight changes in their size and shape across the three cycles. In addition, the repeatability was further supported by the changes in plasmon peak positions observed during the assembly-disassembly cycle. These results indicate that reversible NC formation can be reliably achieved for at least three cycles.

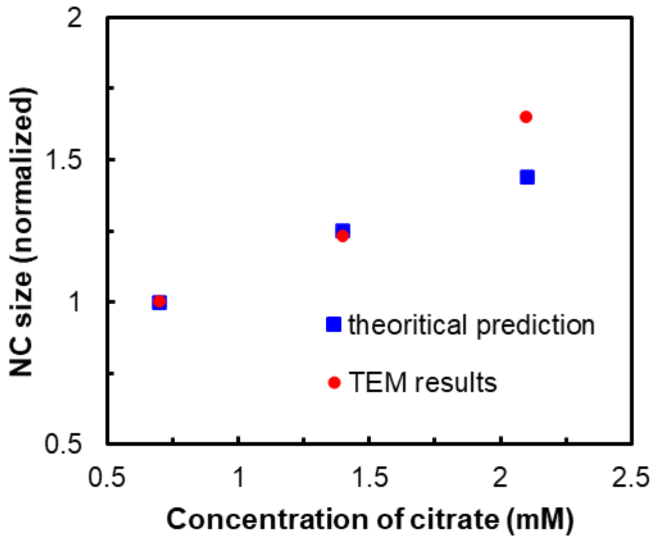


**Figure S9.** Relationship between citrate concentration and NC size. The NC sizes are normalized to the size obtained at 0.70 mM citrate (set as 1). Red circles represent TEM results, and blue squares indicate theoretical predictions.

In Pickering emulsion, the total droplet volume $V$ is proportional to the cube of the droplet diameter ($V \propto D^3$). When the citrate concentration from 0.7 mM to 1.4 and 2.1 mM, the

droplet volume is expected to double and triple, respectively, resulting in sizes approximately 1.25 and 1.44 times larger. The TEM results show good agreement with the theoretical predictions; however, NCs larger than expected were observed at higher citrate concentrations. This deviation suggests a sensitivity of NC size to changes in phase separation equilibrium, as further supported by **Figure S10**.

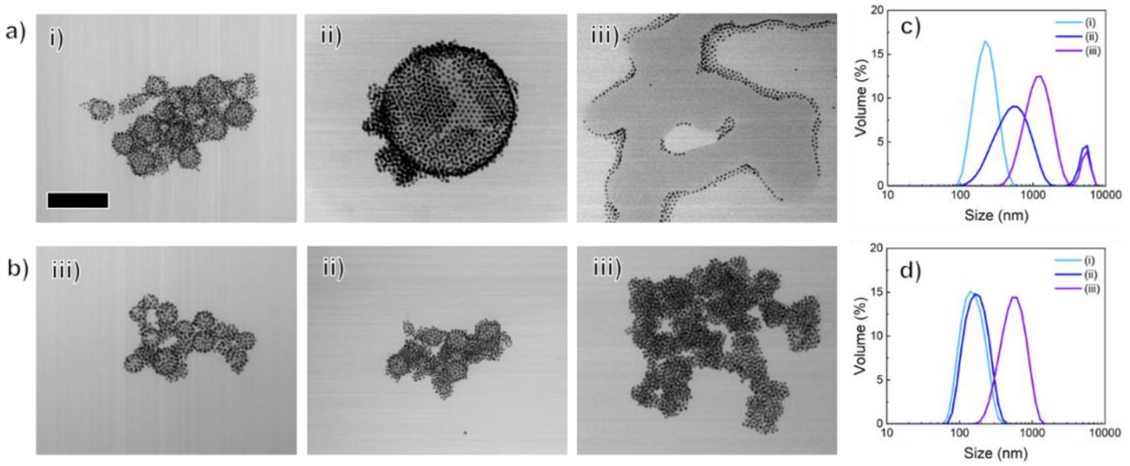


**Figure S10.** TEM images of Au NCs formed in (a) THF and (b) dioxane with different water contents (i) 2%, (ii) 4%, and (iii) 10% in THF and dioxane, respectively. The scale bar in (a-i) corresponds to 200 nm and is common for all images. (c,d) DLS results for (a) and (b).

**Figure S10** shows TEM images and DLS results of Au NCs formed in THF and dioxane at different water contents. In the case of THF, TEM observation confirmed the size of Au NCs increased to about 400 nm at 4% water content, indicating that water content influences the equilibrium of phase separation and NC size. At 10% water content, Au NCs were not observed in TEM images, suggesting a stability limit in NC size. The DLS results also show an increase in aggregate size and a tendency toward aggregation with increasing water content. On the other hand, no significant size change in Au NCs was observed between 2-4% in the case of dioxane. This result is consistent with our expectation that dioxane strongly extracts water and that NC size depends on interface stabilization. When the water content was 10%, aggregates that had lost their hollow structures were observed rather than NCs. This is likely due to the increase in water content, which causes citrate to leach out of the NCs, resulting in structural deflation. This tendency with dioxane was also observed in our previous report.[4]

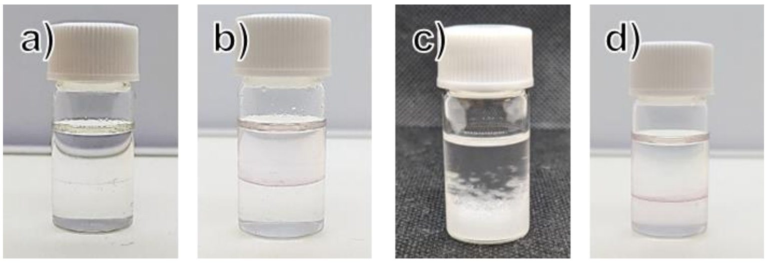


**Figure S11.** The mixture of saturated citric acid aqueous solution and THF (a) without and (b) with OEG-Au NPs. The mixture of saturated citric acid aqueous solution and dioxane (c) without and (d) with OEG-Au NPs.

**Figure S11** shows pictures of THF or dioxane/saturated citrate aqueous mixtures with and without OEG-Au NPs. In the absence of OEG-Au NPs, THF showed phase separation, whereas dioxane showed citrate precipitation. It indicated that dioxane exhibits a higher affinity for water than does THF, leading to citrate precipitation. On the other hand, the dioxane/citrate aqueous interface can be stabilized by OEG-Au NPs without citrate precipitation. This suggests that the stabilization of the interface by OEG-Au NPs allows phase separation to be maintained and Pickering emulsions to form, even in solvents with a high affinity for water.

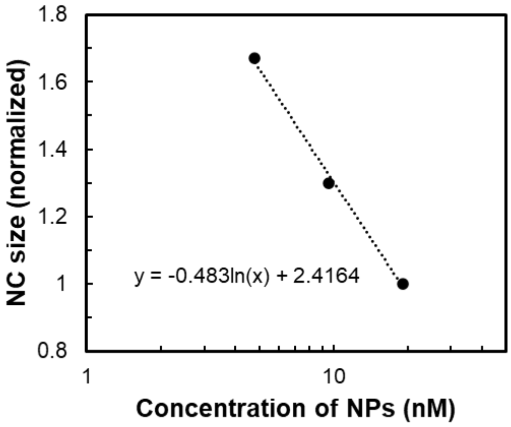


**Figure S12.** Relationship between NP concentration and NC size. The NC sizes are normalized to the size obtained at 19.2 nM NPs (set as 1). The dashed line represents an approximate trend curve.

Empirical studies have shown that droplet diameter $D$ in Pickering emulsions often follows a power-law relationship with the particle concentration $C$ ($D \propto C^{-n}$); where $n$ typically ranges from 0.2 to 0.5, depending on system conditions. From the TEM images (**Figure 3f**), the NC sizes were approximately 1.30- and 1.67-fold larger when the NP concentration was reduced to 1/2 and 1/4 of that in **Figure 3f-i**, respectively. These values closely matched the theoretical prediction for n=0.37 (1.29 and 1.67), suggesting that NC size can be effectively tuned by adjusting NP concentration, as in conventional Pickering emulsions. Indeed, the size ratio exhibited a logarithmic dependence on NP concentration, as shown in **Figure S12**.

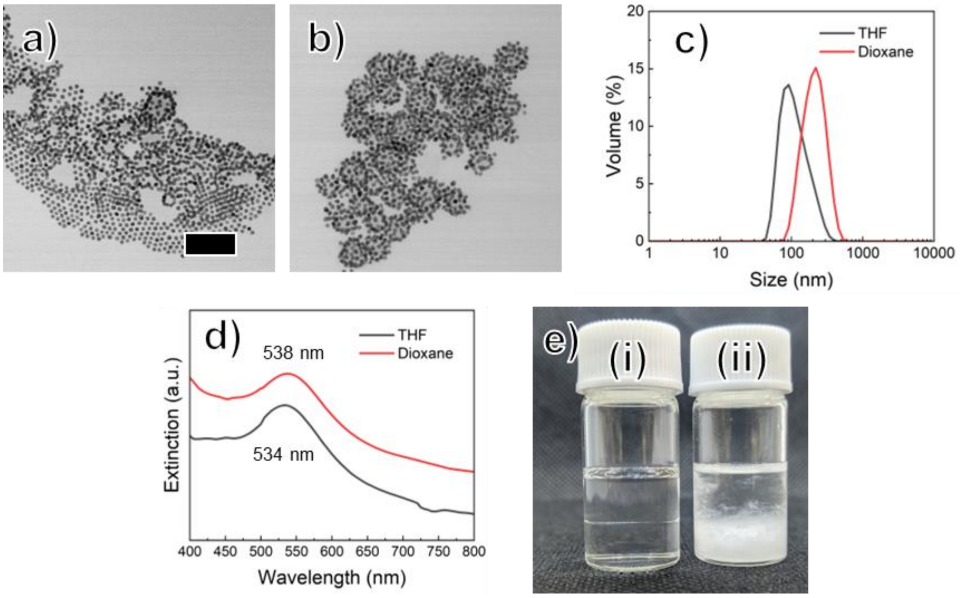


**Figure S13.** TEM images of Au NCs with phosphate formed in (a) THF and (b) dioxane. The scale bar in (a) corresponds to 100 nm and is common for all images. (c) DLS results of (a) and (b). (d) Extinction spectra of samples shown in (a) and (b). (e) Photographs of the mixture of THF and 2 M aqueous solution of (i) sodium citrate and (ii) sodium phosphate.

**Figure S13a-c** shows TEM images and DLS results of Au NCs formed with sodium phosphate instead of sodium citrate in THF and dioxane. In the case of THF, NCs were not observed by TEM observation. On the other hand, some voids were observed, likely representing traces of NC formation, and the formation of NP assemblies was confirmed by DLS. The extinction spectra (**Figure S13d**) are also red-shifted to 534 nm and 538 nm, respectively, compared to the peak position in the dispersed state (523 nm, **Figure S5**), indicating the formation of aggregate structures. These redshifts are smaller than those observed in the citrate system, which is consistent with TEM images showing the formation of smaller NCs. Therefore, it is expected that small NCs form but collapse during the drying process. **Figure S13e** shows the phase separation behavior of THF and an aqueous solution of citrate and phosphate. The citric acid solution exhibited phase separation, whereas the phosphonate solution initially underwent phase separation but rapidly precipitated, similar to the behavior observed with citrate solution and dioxane. Therefore, when phosphonate is used, NC formation is likely governed by interfacial stability, as in the case of dioxane. Thus, NC formation is influenced by the salt species in THF. In contrast, Au NCs were observed in the case of dioxane. This result suggests that NC formation in dioxane is not significantly influenced by the salt type and can occur with salts other than citric acid.

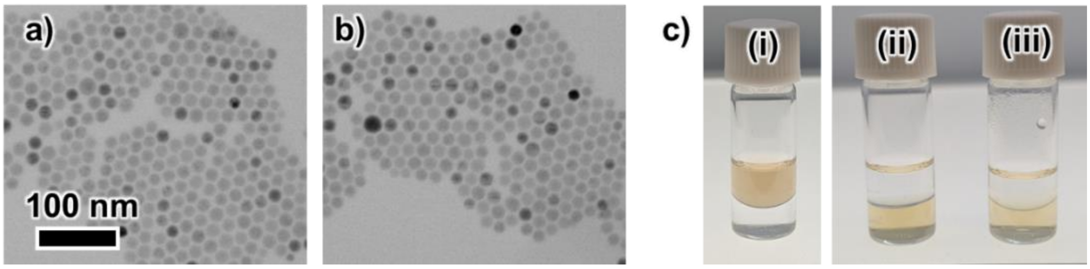


**Figure S14.** TEM images of $Fe_3O_4$ NPs modified with (a) m-EG8C11-$PO_3H_2$ and (b) m-EG9C2-$PO_3H_2$. (c) Dispersibility of $Fe_3O_4$ NPs modified with (i) oleic acid, (ii) m-EG8C11-$PO_3H_2$, and (iii) m-EG9C2-$PO_3H_2$. The upper layer is hexane, and the lower layer is water.

**Figure S14a** and **b** show TEM images of $Fe_3O_4$ NPs modified with m-EG8C11-$PO_3H_2$ and m-EG9C2-$PO_3H_2$. **Figure S14c** shows differences in dispersibility of $Fe_3O_4$ NPs before and after surface modification. Prior to modification, the NPs were dispersed in hexane; however, after OEG modification, they were dispersed in water, indicating successful surface modification.

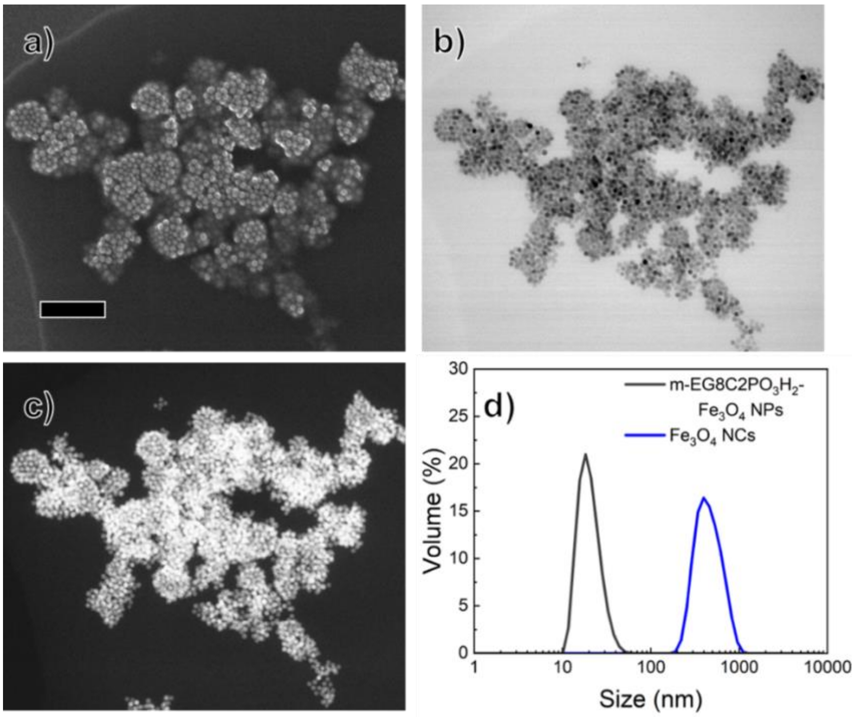


**Figure S15.** (a) SEM, (b) TEM, and (c) HAADF-STEM images of $Fe_3O_4$ NCs from $Fe_3O_4$ NPs modified with m-EG9C2-$PO_3H_2$. The scale bar in (a) corresponds to 200 nm and is common for all images. (d) DLS results before and after $Fe_3O_4$ NC formation when m-EG9C2-$PO_3H_2$ is used as a ligand.

**Figure S15** shows TEM images and DLS results of $Fe_3O_4$ NCs from $Fe_3O_4$ NPs modified with m-EG9C2-$PO_3H_2$. The results indicated that $Fe_3O_4$ NCs were also obtained from $Fe_3O_4$ NPs modified with OEG ligands which have a shorter alkyl domain. The results suggest that the presence of a terminal OEG domain is the most important factor in NC formation and that the molecular structure can be modified to some extent.

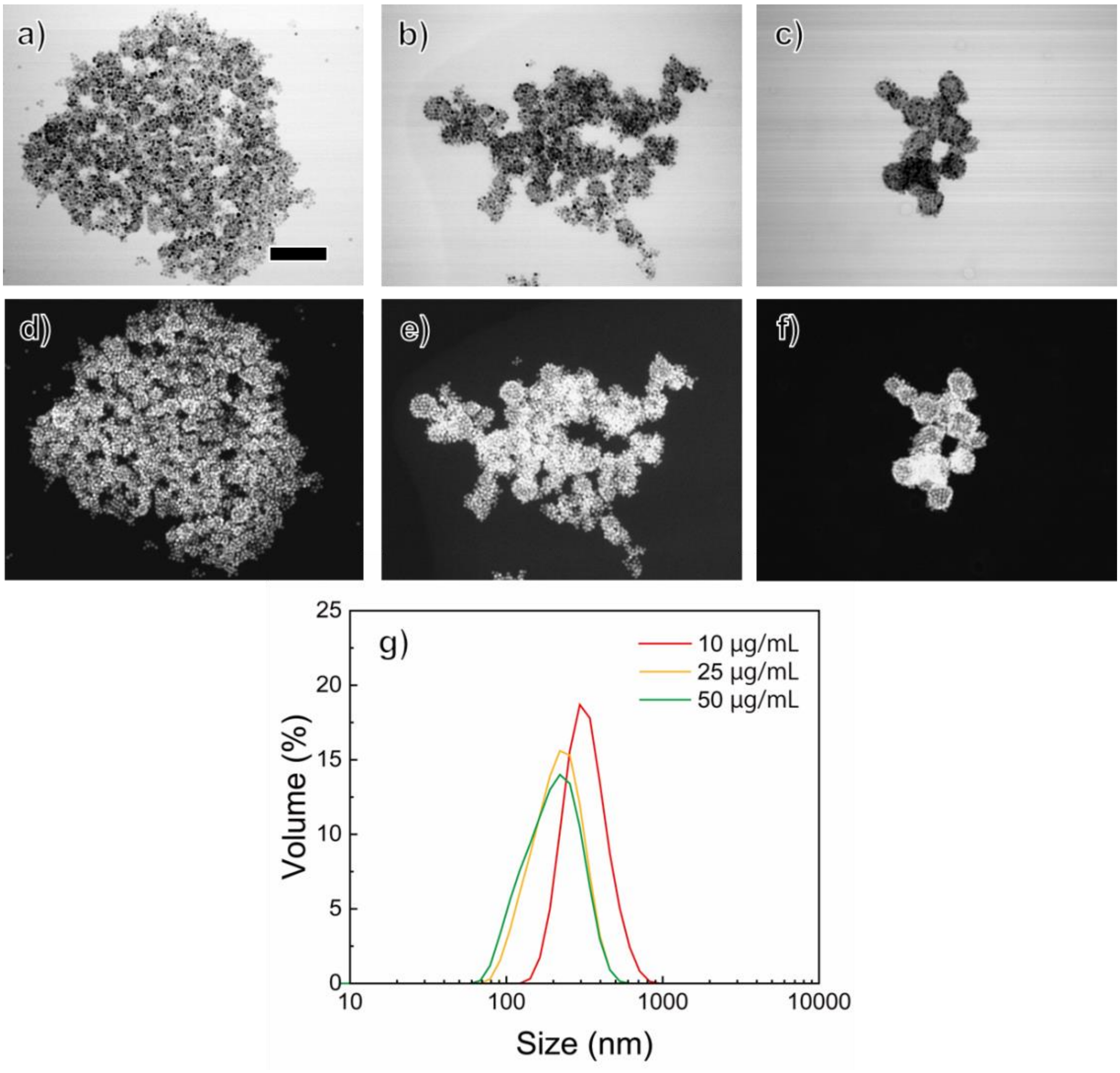


**Figure S16.** TEM images of $Fe_3O_4$ NCs with different concentrations of $Fe_3O_4$ NPs, (a) 50 µg/mL, (b) 25 µg/mL, and (c) 10 µg/mL. (d-f) HAADF-images of (a-c). (g) DLS results of $Fe_3O_4$ NCs with different concentrations of $Fe_3O_4$ NPs. The scale bar is 250 nm and common to all images.

**Figure S16** shows STEM images and DLS results of $Fe_3O_4$ NCs with different concentrations of $Fe_3O_4$ NPs. As the concentration of $Fe_3O_4$ NPs is decreased, the size of $Fe_3O_4$ NCs increases due

to the changes in the balance of the number of NPs and the surface area of the interface. It demonstrated that the size of inorganic NCs can be easily controlled by changing solvent conditions or the concentration of inorganic NPs.

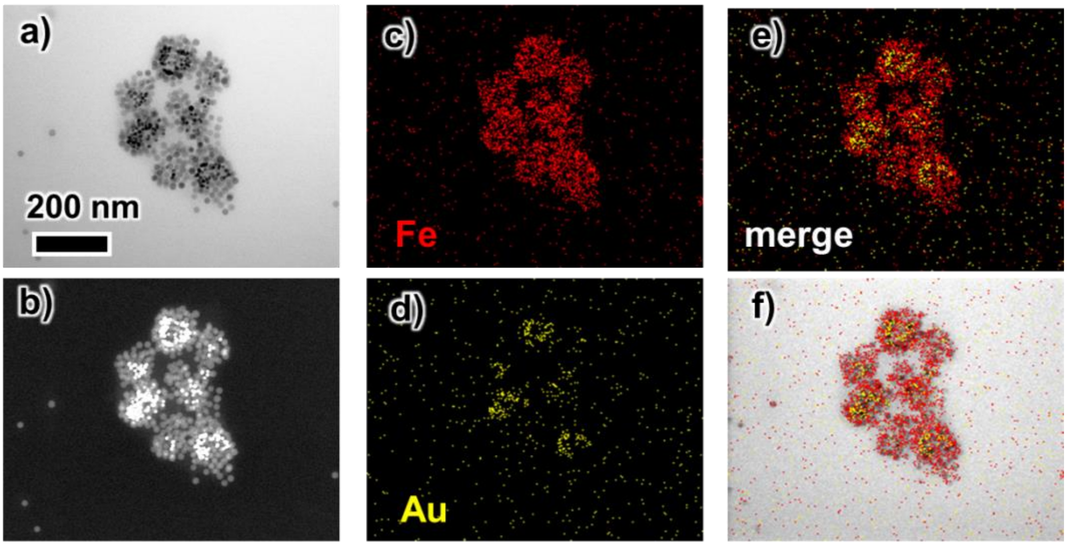


**Figure S17.** (a) TEM and (b) HAADF-STEM images of $Fe_3O_4$ NCs encapsulating 10 nm Au NPs. EDS mapping images for (c) Fe and (d) Au in (a). (e) Merged image of (c) and (d). (f) Merged image of (a) and (e). The scale bar in (a) represents 200 nm and is common for a-f. EDS mapping was conducted using Fe-K and Au-L signals.

**Figure S17** compares TEM, HAADF-STEM, and EDS mapping images. Comparing **Figure S17a** and **Figure S17b**, the smaller size and higher contrast particles in **Figure S17a** appear brighter in HAADF-STEM, suggesting that they are Au nanoparticles with high electron density. This is also consistent with the particle sizes ($Fe_3O_4$: 20 nm, Au: 10 nm). The EDS mapping results further support that the observed differences in particle size and contrast seen in TEM images arise from the distinct identities of $Fe_3O_4$ and Au NPs. Based on these findings, $Fe_3O_4$ and Au NPs were also distinguished in cryo-TEM analysis by differences in particle size and contrast.

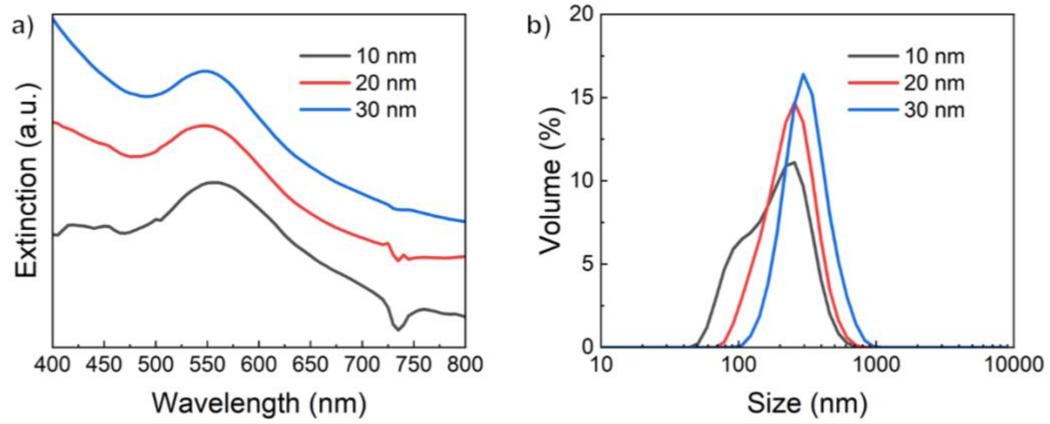


**Figure S18.** (a) Extinction spectra and (b) DLS results of $Fe_3O_4$ NCs encapsulating different sizes of Au NPs

**Figure S18a** shows extinction spectra of $Fe_3O_4$ NCs encapsulating different sizes of Au NPs. The peak positions of $Fe_3O_4$ NCs encapsulating 10, 20, and 30 nm Au NPs were 557 nm, 548 nm, and 547 nm, respectively. In general, Au nanoparticles with larger sizes show longer wavelength plasmon peaks, but the opposite trend was observed for $Fe_3O_4$ NCs encapsulating Au NPs. This result is consistent with the STEM observations, which suggest that the Au NPs are encapsulated more in the capsule, causing plasmon coupling. **Figure S18b** shows DLS results. Although a trend toward smaller $Fe_3O_4$ NC size with smaller Au NPs was observed in the DLS, no significant change in size was observed. In addition, a shoulder peak was observed when 10 nm Au NPs were used. This would be due to the formation of small $Fe_3O_4$ NCs with the particles as their core. In fact, STEM observations show the formation of small $Fe_3O_4$ NCs encapsulating several Au NPs when using 10 nm Au NPs.

**Movie S1** and **S2** show the continuous tilt series and XY slice movies of Au NCs reconstructed from cryo-ET. **Movie S3** and **S4** show the continuous tilt series and XY slice movies of $Fe_3O_4$ NCs encapsulating Au NPs reconstructed from cryo-ET.
**Movies S1-S4**. ... Gold-sputtered holey carbon film was used for sample support.

REFERENCE

[1] D. N. Mastronarde, *J. Struct. Biol.* **2005**, *152*, 36–51.
[2] A. Xu, C. Xu, *Bioengineering* **2021**.
[3] J. R. Kremer, D. N. Mastronarde, J. R. McIntosh, *J. Struct. Biol.* **1996**, *116*, 71–76.
[4] J. Wei, K. Niikura, H. Mitomo, Y. Mastuo, K. Ijiro, *J. Nanosci. Nanotechnol.* **2017**, *17*, 9149–9156.
